# Neocortex- and hippocampus-specific deletion of *Gabrg2* causes temperature-dependent seizures in mice

**DOI:** 10.1038/s41419-021-03846-x

**Published:** 2021-05-28

**Authors:** Xinxiao Li, Shengnan Guo, Siying Xu, Zhangping Chen, Lei Wang, Jiangwei Ding, Junming Huo, Lifei Xiao, Zhenquan He, Zhe Jin, Feng Wang, Tao Sun

**Affiliations:** 1 Ningxia Key Laboratory of Cerebrocranial Disease, The Incubation Base of National Key Laboratory, Ningxia Medical University, Yinchuan, Ningxia China; 2 Department of Neurosurgery, The Fifth Affiliated Hospital of Zhengzhou University, Zhengzhou, Henan China; 3 Department of Neurosurgery, General Hospital, Ningxia Medical University, Yinchuan, Ningxia China; 4 Department of Medical Cell Biology, Uppsala University, Uppsala, Uppsala, Sweden; 5 Department of Neurosurgery, The First Affiliated Hospital, Zhejiang University School of Medicine, Hangzhou, Zhejiang China

**Keywords:** Epilepsy, Genetics of the nervous system

## Abstract

Mutations in the *GABRG2* gene encoding the γ-aminobutyric acid (GABA) A receptor gamma 2 subunit are associated with genetic epilepsy with febrile seizures plus, febrile seizures plus, febrile seizures, and other symptoms of epilepsy. However, the mechanisms underlying *Gabrg2*-mediated febrile seizures are poorly understood. Here, we used the Cre/loxP system to generate conditional knockout (CKO) mice with deficient *Gabrg2* in the hippocampus and neocortex. Heterozygous CKO mice (*Gabrg2*^*fl/wt*^*Cre*^*+*^) exhibited temperature-dependent myoclonic jerks, generalised tonic-clonic seizures, increased anxiety-like symptoms, and a predisposition to induce seizures. Cortical electroencephalography showed the hyperexcitability in response to temperature elevation in *Gabrg2*^*fl/wt*^*Cre*^+^ mice, but not in wild-type mice. *Gabrg2*^*fl/wt*^*Cre*^*+*^ mice exhibited spontaneous seizures and susceptibility to temperature-induced seizures. Loss of neurons were observed in cortical layers V–VI and hippocampus of *Gabrg2*^*fl/wt*^*Cre*^*+*^ mice. Furthermore, the latency of temperature- or pentylenetetrazol-induced seizures were significantly decreased in *Gabrg2*^*fl/wt*^*Cre*^*+*^ mice compared with wild-type mice. In summary, *Gabrg2*^*fl/wt*^*Cre*^*+*^ mice with *Gabrg2* deletion in the neocortex and hippocampus reproduce many features of febrile seizures and therefore provide a novel model to further understand this syndrome at the cellular and molecular level.

## Introduction

Genetic epilepsy with febrile seizures plus (GEFS + ) is a well-known familial epileptic syndrome of childhood with autosomal dominant trait^1^. Its phenotype ranges from most common febrile seizures (FS) to febrile seizures plus (FS + ) and absences, and myoclonic seizures, or Dravet syndrome^1–4^. Genes encoding for voltage-gated sodium channels^5–7^ and γ-aminobutyric acid (GABA) type A receptors (GABA_A_Rs) are involved in GEFS + aetiology. Among them, *GABRG2*, which encodes the GABA_A_R γ2 subunit, has been identified as a causal gene for GEFS +. Different mutation types in *Gabrg2* have been reported in GEFS + families, including missense^8–15^, nonsense^16–19^, frameshift^15,20^, and splice site mutations^21,22^. These mutations result in dominant-negative suppression of the remaining GABA_A_R function^23^ and cellular toxicity^24^.

GABA_A_R are pentameric ligand-gated ionotropic chloride (Cl^−^) channels that are ubiquitously expressed in the central nervous system. They mediate the majority of fast inhibitory neurotransmission, play a fundamental role in restraining and sculpting neuronal activity, and have been implicated in animal models of seizures^25,26^. There are 19 GABA_A_R subunits (α1–6, β1–3, γ1–3, δ, ε, θ, π, and ρ1–3) with the most prevalent synaptic isoform comprising two α1, two β2, and one γ2 subunit^27,28^. The diversity of GABA_A_R subunits affects the localisation, pharmacological properties, and function of assembled receptors^29–31^. The γ2 subunit is required for postsynaptic localisation and clustering of GABA_A_R^32,33^. *GABRG2* mutations in epilepsy target different functional domains and influence GABA_A_R gating, membrane trafficking, and clustering at synapses^11,34^. Most mutations are not fully penetrant, and the intrafamilial phenotype varies considerably, indicating that other modifying genes may exist and influence the development and persistence of epilepsy^35,36^. The mechanisms linking *GABRG2* mutations with pathophysiological variations of GEFS + remain unknown.

*Gabrg2* loss-of-function mouse models exhibit different epilepsy phenotypes. Heterozygous mice with a targeted deletion of *Gabrg2* (*Gabrg2*^*+/-*^) have absence seizures but no spontaneous generalised tonic-clonic seizures (GTCS). Heterozygous *Gabrg2*^*+/Q390X*^ knock-in mice with a nonsense mutation Q390X display GTCS and higher mortality^24,37^. It is difficult to develop an appropriate experimental model that adequately reproduces the functional alterations evidenced by mutations in GEFS + patients.

Here, we generated a novel conditional knockout (CKO) mouse model with *Gabrg2* deletion in the neocortex and hippocampus using the Cre/loxP system. We recorded the seizure occurrence and cortical electroencephalography (EEG) activity at different temperatures in heterozygous mice. Our results demonstrate that this model reproduces many features of human GEFS +, and aids in understanding the seizure-related pathologic features of this syndrome at the molecular and cellular level.

## Results

### Generation of *Gabrg2*-floxed mice

**Fig. 1:**
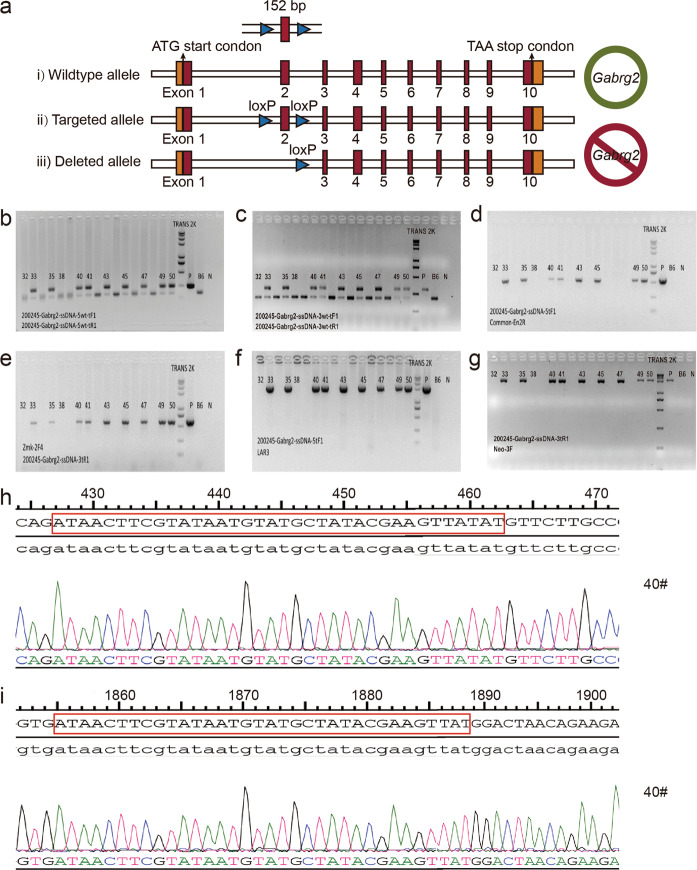
Generation of *Gabrg2*-floxed mice. **a** The transcript *Gabrg2-003* (ENSMUST00000070735.9) was taken as an example to describe the strategy. *Gabrg2* gene has 10 exons, with the ATG start codon in exon 1 and TAA stop codon in exon10. *loxP* sites were inserted in introns 1–2 and introns 2–3 by donor mediated homologous recombination. Exon 2 was floxed by *loxP* sites and can be removed via crossing with Cre-driver lines. Frameshift caused by indel mutations can destroy the *Gabrg2* gene product. **b** *Gabrg2*-floxed heterozygotes or homozygotes were identified by PCR screening of tail-derived genomic DNA using the primer of 5′ preliminary screening probe, and the 158 bp or 248 bp PCR product was the wild type or homozygotes, respectively. The 158 bp and 248 bp bands present heterozygous. **c** The primer of 3’ preliminary screening probe was used for detecting heterozygous or homozygous *Gabrg2*-floxed mice. The 294 bp and 201 bp bands present heterozygous. **d** The *loxP* site and 5’ homologous arm of 5’ ssDNA were detected by D5-5 primer, and a 534 bp band was a positive clone containing the *loxP* site. **e** The *loxP* site and 3’ homologous arm of 3’ ssDNA were detected by D3-3 primer, and the 588 bp band was a positive clone. **f** The *loxP* site and 5’ homologous arm of 3’ ssDNA were also detected by D3-5 primer, and the 1 962 bp band was a positive clone. **g** The *loxP* site and 3’ homologous arm of 5’ ssDNA were detected by D5-3 primer, the 2 013 bp band was a positive clone. **h** PCR product sequencing showed that the *loxP* site was correctly inserted in introns 1-2. **i** Sequencing results indicated that the *loxP* site was correctly inserted in introns 2-3. TRANS 2 K Plus II maker size: 8 000, 5 000, 3 000, 2 000, 1 000, 750, 500, 250, and 100 bp; P: positive control; B6: negative control of which the template is the genomic DNA of C57BL/6 J mice; N: blank control without template.

**Fig. 2:**
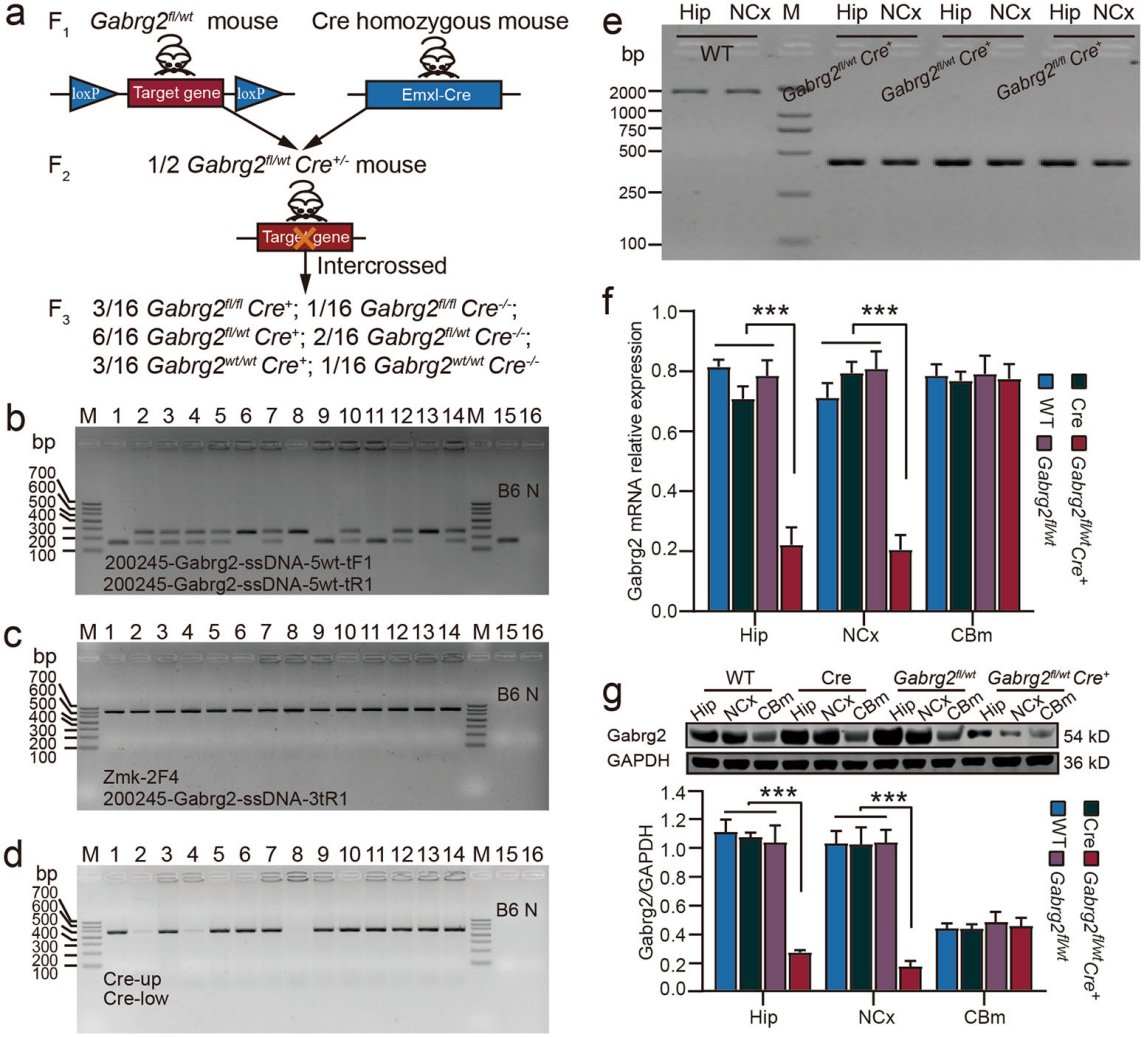
Generation of conditional hippocampus- and neocortex-specific *Gabrg2* knockout mice. **a** Schematic representation of the breeding design to obtain conditional deletion of *Gabrg2* in the neurons of the mice neocortex and hippocampus. This breeding strategy can produce six different genotypes, and the genotype of *Gabrg2*^*fl/wt*^*Cre*^*+*^ mice was screened for experiment. **b** Agarose gel electrophoresis was used to analyses the PCR products. Primer No. 1 was used to detect the heterozygous or homozygous similar to the 5’ preliminary screening probe. **c** The *loxP* site of 3’ ssDNA was detected by primer No. 2, and the 588 bp band was a positive clone containing *loxP*. **d** Primer No. 3 was used to confirm that the Emxl-Cre. Cre-positive mice had a 481 bp band, while the negative did not. The neocortex and hippocampus DNA were extracted from mice No. 3, 5, 6 and 15. **e** Verification of the flox region deletion in the neocortex and hippocampus of *Gabrg2* CKO mice. *Gabrg2* CKO mice had a 424 bp band, whereas the WT mice had a 1 669 bp band. **f** RT-qPCR verified the Gabrg2 mRNA expression level in the hippocampus, neocortex, and cerebellum. The level of Gabrg2 mRNA in neocortex and hippocampus was significantly decreased in *Gabrg2*^*fl/wt*^*Cre*^*+*^ mice (*n* = 6), while there was no significant change in WT (*n* = 6), Cre (*n* = 6), and *Gabrg2*^*fl/wt*^ (*n* = 6) mice. There was no significant difference in the cerebellum between the four groups. **g** Gabrg2 protein expression levels in the hippocampus, neocortex, and cerebellum of the *Gabrg2*^*fl/wt*^*Cre*^*+*^ (*n* = 6), *Gabrg2*^*fl/wt*^ (*n* = 6), Cre^+/+^ (*n* = 6), and WT (*n* = 6) mice. *Gabrg2*^*fl/wt*^*Cre*^*+*^ mice expressed ~40% Gabrg2 protein in the neocortex and hippocampus compared to the WT, Cre, and *Gabrg2*^*fl/wt*^ mice, while there was no significant change in the cerebellum among the four groups. M: marker; B6: negative control of which the template is the genomic DNA of C57BL/6 J mice; N: blank control without template; Hip: hippocampus; NCx: neocortex; CBm: cerebellum. One-way ANOVA with Bonferroni *post hoc* test; ***P < 0.001 vs WT, *t*-test (two-tailed).

We designed the *Gabrg2* gene knockout targeting strategy as shown in Fig. 1a. The schematic design of primers for *Gabrg2* flox mice identification is shown in Fig. S1. Primer pair No. 1 was used to detect the wild type (WT) and test whether the *loxP* site was inserted correctly. The results of agarose gel electrophoresis showed the presence of a 158 bp single PCR (WT), 248 bp single PCR (*loxP* homozygote), and 248 and 158 bp PCR bands (*loxP* heterozygote) (Fig. 1b). When using primer pair No. 2, the presence of 294 bp band indicated a positive clone (Fig. 1c). The 534 bp PCR product was a positive clone containing the *loxP* of 5’ ss-DNA and 5’ homologous arm using No. 3 (Fig. 1d), whereas the 588 bp PCR product was a positive clone containing the *loxP* of 3’ ss-DNA and 3’ homologous arm using primer pair No. 4 (Fig. 1e). The results of agarose gel electrophoresis of using primer pairs No. 5 and No. 6 indicated that the presence of 1 962 (Fig. 1f) and 2 013 bp (Fig. 1g) bands were positive clones. Primers are presented in Supplemental Tables S1. Mice (No. 40, 41, 45, 49, and 50) were deemed to be chimeric mice with the *Gabrg2*^*fl/wt*^ genotype. The PCR products of mice No. 40, 41, 45, and 49 were sequenced (No. 50 mouse died of weakness), and the results showed the correct insertion of *loxP* sites (Fig. 1h, i). All insertion sites were confirmed by PCR and sequencing, indicating the successful generation of *Gabrg2*-floxed mice.

### Generation of conditional hippocampus- and neocortex-specific *Gabrg2* knockout mice

*Gabrg2*^*fl/wt*^*Cre*^*+*^ CKO mice were produced by crossing F_1_ *Gabrg2*-floxed mice with Emx1-IRES-Cre mice containing the Emx1 locus that drives the expression of Cre recombinase to most neurons in the hippocampus and neocortex. The F_2_ mice were intercrossed to generate F_3_ mice (Fig. 2a.) Based on the results of agarose gel electrophoresis (248 and 158 bp PCR products corresponding to correct 3’ *loxP* site and positive Cre, respectively), the *Gabrg2*^*fl/wt*^*Cre*^*+*^ were identified and used for seizure phenotype testing (Fig. 2b, c and d). We performed PCR using the DNA extracted from the mouse hippocampus and neocortex to determine whether *Gabrg2* in the brain tissues was deleted. *Gabrg2*^*fl/wt*^*Cre*^*+*^ mice had a 424 bp PCR product, whereas WT mice had a 1 669 bp (Fig. 2e). Primers are presented in Supplemental Tables S2.

We performed RT-qPCR and western blot to detect the mRNA and protein expression levels, respectively, of the γ2 subunit in the hippocampus and neocortex. *Gabrg2*^*fl/wt*^*Cre*^*+*^ mice were normally viable and showed ~40% reduction in γ2 mRNA (Fig. 2f) and protein (Fig. 2g) levels in the neocortex and hippocampus, but not in the cerebellum, compared to WT, Cre, and *Gabrg2*^*fl/wt*^ mice. Our results confirmed the deletion of *Gabrg2* from the neocortex and hippocampus of the *Gabrg2*^*fl/wt*^*Cre*^*+*^ mice.

### General phenotypic observations of *Gabrg2*^*fl/wt*^*Cre*^*+*^ mice

**Fig. 3:**
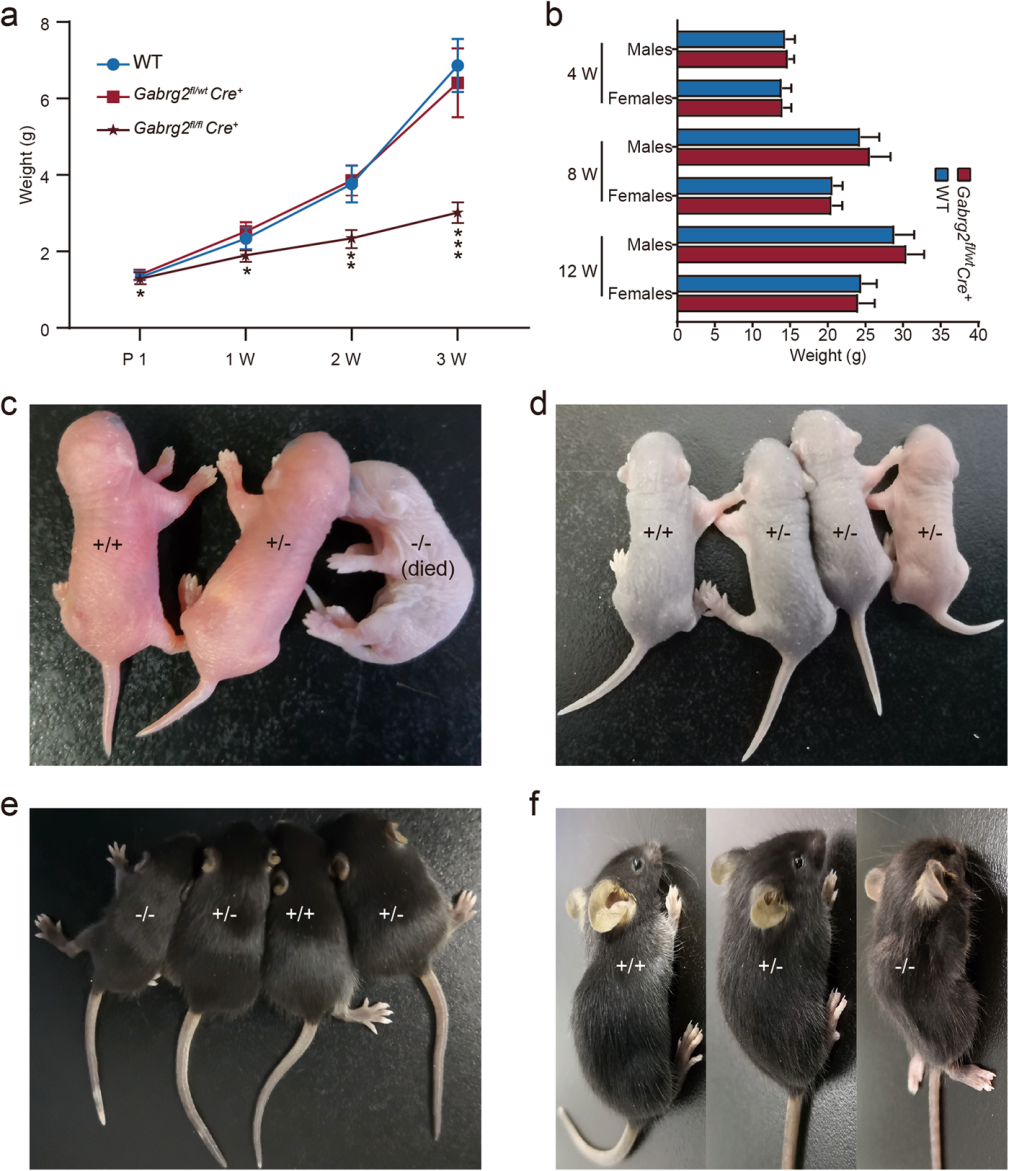
Gabrg2 deletion affects the body weight and defective development. **a** The line graph summarising the average body weight of *Gabrg2*^*fl/fl*^*Cre*^*+*^, *Gabrg2*^*fl/wt*^*Cre*^*+*^, and WT mice at postnatal 1 day (*Gabrg2*^*fl/fl*^*Cre*^*+*^, *n* = 33; *Gabrg2*^*fl/wt*^*Cre*^*+*^, *n* = 46; WT, *n* = 41), 1 week (*Gabrg2*^*fl/fl*^*Cre*^*+*^, *n* = 21; *Gabrg2*^*fl/wt*^*Cre*^*+*^, *n* = 46; WT, *n* = 41), 2 weeks (*Gabrg2*^*fl/fl*^*Cre*^*+*^, *n* = 16; *Gabrg2*^*fl/w*^*tCre*^*+*^, *n* = 46; WT, *n* = 41), and 3 weeks (*Gabrg2*^*fl/f*^*lCre*^*+*^, *n* = 9; *Gabrg2*^*fl/wt*^*Cre*^*+*^, *n* = 46; WT, *n* = 41) postnatal. The body weight of the *Gabrg2*^*fl/fl*^*Cre*^*+*^ mice was lower than *Gabrg2*^*fl/wt*^*Cre*^*+*^ and WT mice. **b** A bar graph summarising the average body weight of *Gabrg2*^*fl/wt*^*Cre*^*+*^ and WT mice (*n* = 13 for each group) at postnatal 4 weeks, 8 weeks, and 12 weeks. There was no significant difference in body weight between mice of the same sex and at the same growth stage (4 weeks, 8 weeks, and 12 weeks) in the *Gabrg2*^*fl/wt*^*Cre*^*+*^ and WT groups. **c** The morphology of *Gabrg2*^*fl/fl*^*Cre*^*+*^, *Gabrg2*^*fl/wt*^*Cre*^*+*^, and WT mice from first day, one week (**d**), two weeks (**e**), and three weeks (**f**) after birth. *Gabrg2*^*fl/fl*^*Cre*^*+*^ mice died within the first day after birth. As the development progresses, homozygous mice are getting thinner in shape, especially in three weeks. One-way ANOVA with Tukey’s multiple comparisons test; *P < 0.05, **P < 0.01 and ***P < 0.001 vs WT, *t*-test (two-tailed).

Most (24/33) of the homozygous mice (*Gabrg2*^*fl/fl*^*Cre*^*+*^) died within a few days after birth, and few mice (9/33) survived for 3 weeks and up to 4 weeks. These homozygous mice had lower birth weight (*n* = 33) than the heterozygous (*n* = 46) and WT (*n* = 41, *P* = 0.042) mice at one day of age, while there was no significant difference between heterozygous and WT mice (*P* = 0.74) (Fig. 3a). The weight of homozygous mice was markedly reduced with age, especially at 3 weeks (*P* = 0.00037). There was no significant difference in body weight between *Gabrg2*^*fl/wt*^*Cre*^*+*^ and WT mice at 1, 2, and 3 weeks of age (*P* > 0.05). There was no significant difference in body weight between *Gabrg2*^*fl/wt*^*Cre*^*+*^ and WT mice during subsequent development (Fig. 3b). The few γ2 homozygous survivors exhibited motor deficits, impaired grasping and righting reflex, and abnormal gait. Excessive hyperactivity was not observed in *Gabrg2*^*fl/fl*^*Cre*^*+*^ mice. The gross morphology of mice with different genotypes was shown at postnatal 1 day (P1, Fig. 3c), 1 week (P7, Fig. 3d), 2 weeks (P14, Fig. 3e), and 3 weeks (P21, Fig. 3f). These results suggest that *Gabrg2* may play a crucial role during development.

### Reduced Gabrg2 expression promoted neuronal damage in the hippocampus and neocortex of the *Gabrg2*^*fl/wt*^*Cre*^*+*^ mice

**Fig. 4:**
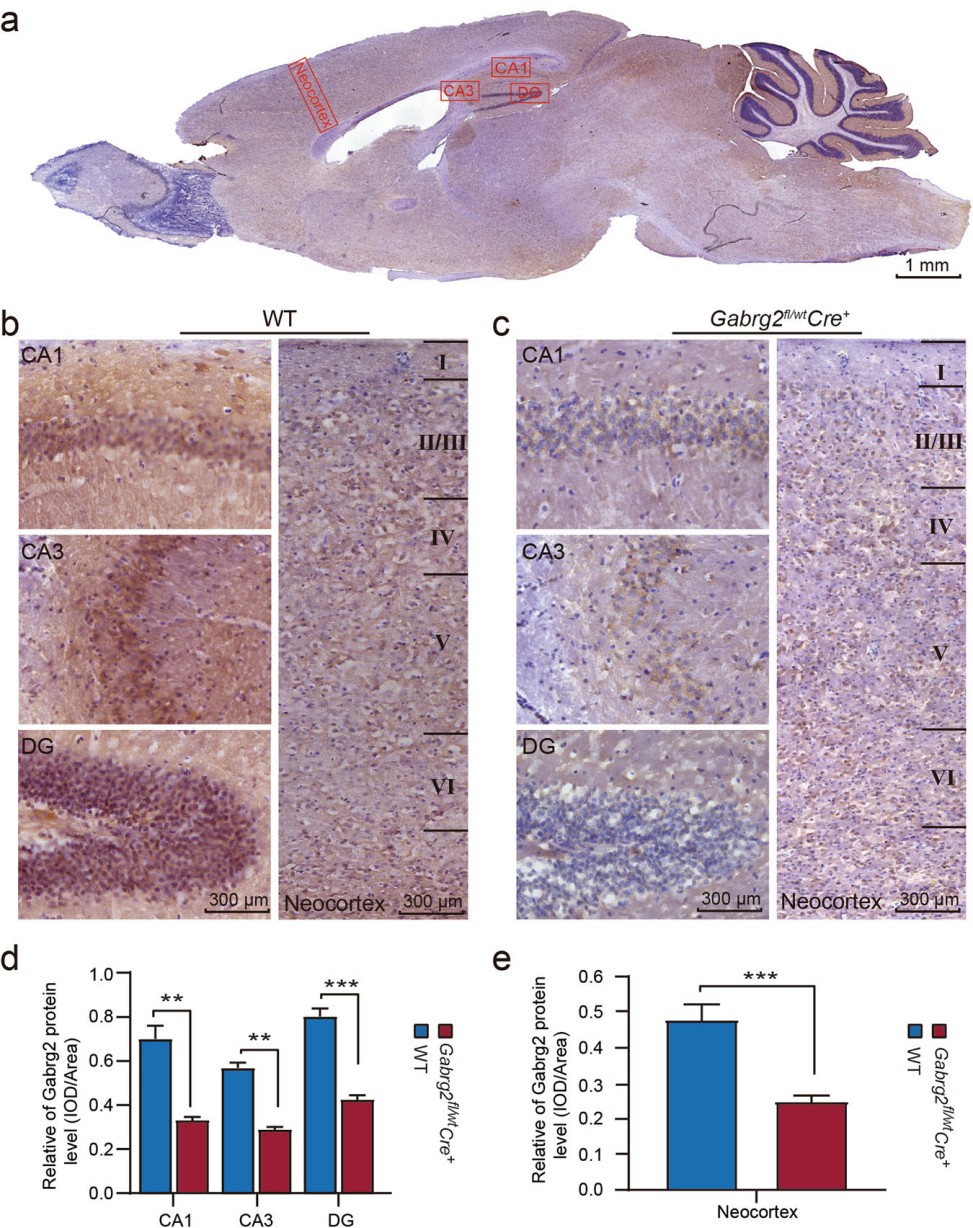
Immunohistochemistry of neurons in the mouse brain. **a** The immunohistochemistry of the *Gabrg2*^*fl/wt*^*Cre*^*+*^ mice brain was performed. The CA1, CA3, and DG, and neocortex regions were selected in a sagittal section. This mouse brain section was immunostained with an anti-Gabrg2 antibody, showing brown stain. Scale bar: 1 mm. **b** Representative immunohistochemical staining of the hippocampus and cerebral cortex of WT mice using the antibody against Gabrg2, where most of neurons in CA1, CA3 and DG, and the neocortex regions were stained. Compared with *Gabrg2*^*fl/wt*^*Cre*^*+*^ mice, neurons in the hippocampus and neocortex organised in groups in the WT are neat, relatively close, and have a lot of synapses. Scale bar: 300 µm. **c** Representative immunohistochemistry images showing the Gabrg2 proteins in the CA1, CA3, and DG, and neocortex regions of *Gabrg2*^*fl/wt*^*Cre*^*+*^ mice. The density of stained neurons was significantly lower than that of the control group. Scale bar: 300 µm. **d** Histograms of the Gabrg2 protein expression in the hippocampus (CA1, CA3 and DG) quantified from immunohistochemistry analysis in WT (*n* = 4) and *Gabrg2*^*fl/wt*^*Cre*^*+*^ mice (*n* = 6). **e** The relative Gabrg2 protein level in the neocortex of WT and *Gabrg2*^*fl/wt*^*Cre*^*+*^ mice (*n* = 4 for WT and *n* = 6 for KO mice). Data shown are mean ± standard error of mean. **P < 0.01 and ***P < 0.001 vs WT, *t*-test (two-tailed).

Mouse sagittal brain sections were used for Gabrg2 immunohistochemistry (Fig. 4a). Gabrg2 immunoreactivity in WT mice was mainly detected in the hippocampus and cerebellum, followed by the cortex, thalamus, and hypothalamus; the weakest staining was observed in the olfactory bulb. These data indicate that the expression of Gabrg2 varies among different brain sub-regions (Fig. 4b). A dramatic decrease of Gabrg2 immunoreactivity was observed in *Gabrg2*^*fl/wt*^*Cre*^*+*^ mice, mainly in the hippocampus and neocortex (Fig. 4c), compared with WT mice (Fig. 4b). Quantitative analysis revealed that the Gabrg2 staining intensity was significantly reduced in the hippocampus (CA1, CA3, and DG regions) (Fig. 4d) and neocortex (Fig. 4e) in the *Gabrg2*^*fl/wt*^*Cre*^*+*^ mice (*n* = 6) as compared with the WT mice (*n* = 4). In contrast, the Gabrg2 staining intensity did not differ in the olfactory bulb and brain stem between the two groups (data not shown).

**Fig. 5:**
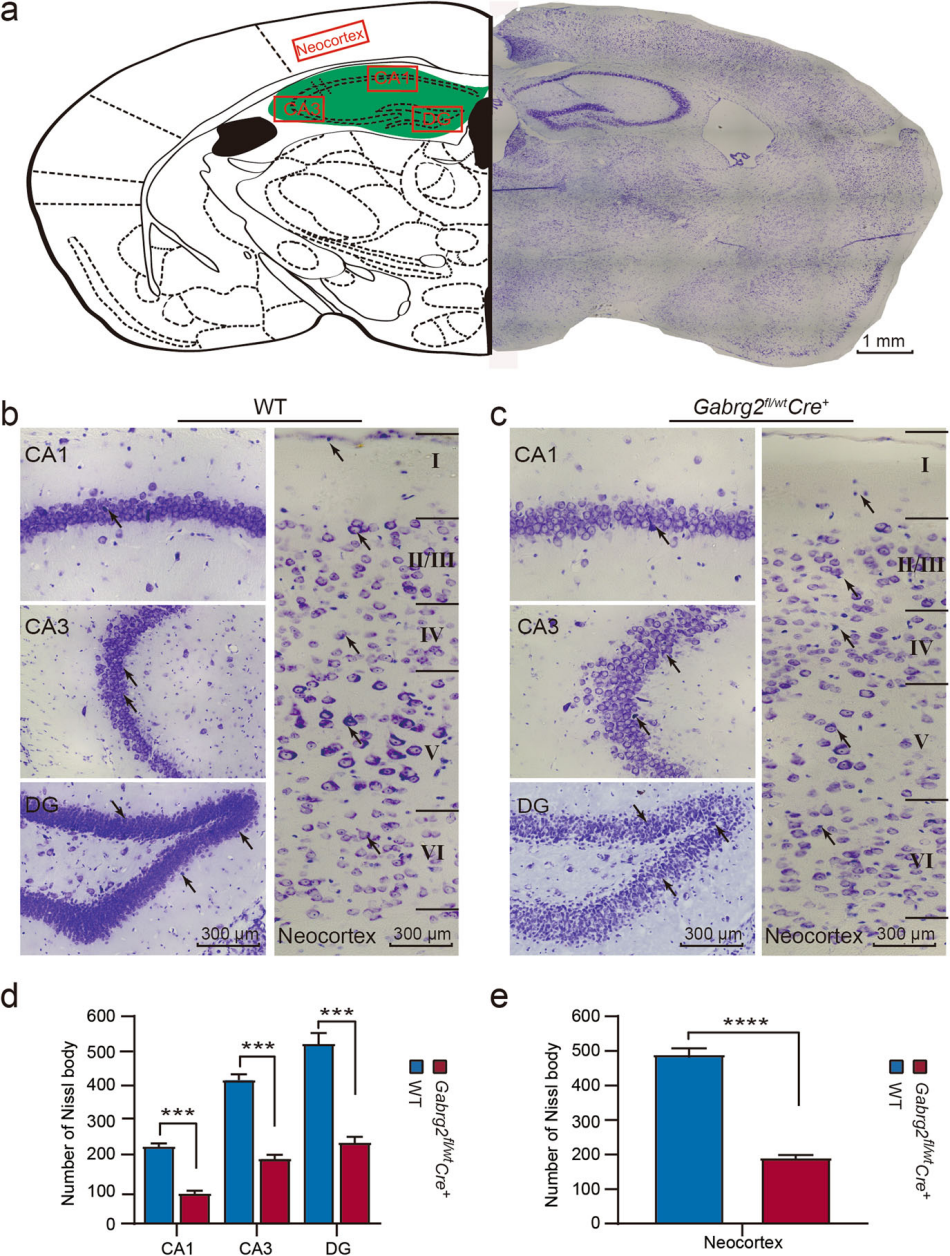
Nissl staining was used to detect the effect of *Gabrg2* deletion on hippocampus and cortical neurons. **a** Comparison of the position and mapping of the left hemisphere. On the right, a representative Nissl image from three independent experiments of the mice brain (coronal section, −1.8 mm relative to the bregma) shows the stained neurons of WT mice. Scale bar: 1 mm. **b** Representative Nissl staining of the hippocampus and cerebral cortex of WT mice. These neurons are neat, relatively close, and have a lot of synapses. In WT mice, layer V (deep Pyramidal) contains the largest pyramidal neurons of the cortex, which project their axons to a variety of cortical and sub-cortical targets. Scale bar: 300 µm. **c** On the sections of the hippocampus’ CA1, CA3 and DG regions, and neocortex in *Gabrg2*^*fl/wt*^*Cre*^*+*^ mice, there is a decrease in the number of neurons, and cells are arranged disorderly. In layer V of the neocortex, the pyramidal neurons were significantly less in the *Gabrg2*^*fl/wt*^*Cre*^*+*^ mice than that of the WT mice. Scale bar: 300 µm. **d** Histograms of the Nissl body counts in the hippocampus quantified from Nissl staining analysis in WT (*n* = 4) and *Gabrg2*^*fl/wt*^*Cre*^*+*^ mice (*n* = 6). **e** The number of Nissl body was counted in the neocortex in two group (*n* = 4 for WT and *n* = 6 for KO). Black arrow: Nissl body. Data shown are mean ± standard error of mean. ***P < 0.001 and ****P < 0.0001 vs WT, *t*-test (two-tailed).

Nissl staining was performed on coronal slices of mouse brains (Fig. 5a), which focused primarily on the CA1, CA3, and DG regions of the hippocampus and on the neocortex (Fig. 5b and c). Neuronal loss was observed in the hippocampus and cortex regions in *Gabrg2*^*fl/wt*^*Cre*^*+*^ (*n* = 6) compared to WT mice (*n* = 4) (Fig. 5d and e), mainly in the CA3 and cortical layers V–VI. These data suggest that *Gabrg2* deletion exacerbates neuronal damage, in the hippocampus and neocortex and may be related to the occurrence of seizures.

### Electrode implantation, spontaneous seizures, and EEG recording in *Gabrg2*^*fl/wt*^*Cre* + *mice*

The electrode implantation surgery was presented in Supplemental Fig. S2a–f. Nine out of 21 adult *Gabrg2*^*fl/wt*^*Cre*^*+*^ mice (8–16 weeks) had spontaneous severe myoclonic jerks (MJs) and GTCSs with an average duration of 68.40 ± 10.50 s (range: 10–120 s) in C57BL/6 J background (Video 1).

We recorded EEGs from adult *Gabrg2*^*fl/wt*^*Cre*^*+*^ mice, and the seizure-related behaviours and baseline EEGs were captured with video monitoring. The baseline EEG at normal body temperature is shown in Fig. S3a. The 4–7 Hz spike-wave discharges (SWDs) associated with seizures or without abnormal behaviour were abnormal EEG activity. Dense epileptiform discharges were detected during GTCS events, and high amplitude spikes (over 60 μV) were recorded in MJs (Fig. S3b, black arrow). The estimated seizure frequency (including MJs and GTCSs) for the *Gabrg2*^*fl/wt*^*Cre*^*+*^ mice was 1.56 ± 0.53 seizures/mouse/day (range: 0–2 seizures; *n* = 9), while the WT mice had no seizures (*n* = 12) (Fig. S3c). The average seizure duration was higher in *Gabrg2*^*fl/wt*^*Cre*^*+*^ mice than in the WT mice (*P* < 0.0001) (Fig. S3d). The number of spikes was significantly higher in the *Gabrg2*^*fl/wt*^*Cre*^*+*^ mice (*n* = 9) than in the WT mice (*n* = 12) (*P* < 0.0001) (Fig. S3e) during a seizure episode. *Gabrg2*^*fl/wt*^*Cre*^*+*^ mice had a higher average spike frequency compared with WT mice (*P* < 0.01) (Fig. S3f). These data indicate that *Gabrg2* deletion increased susceptibility to seizures and suggest that *Gabrg2* plays a crucial role in genetic epilepsy.

### *Gabrg2*^*fl/wt*^*Cre*^*+*^ mice exhibited increased susceptibility to temperature-induced seizures

**Fig. 6:**
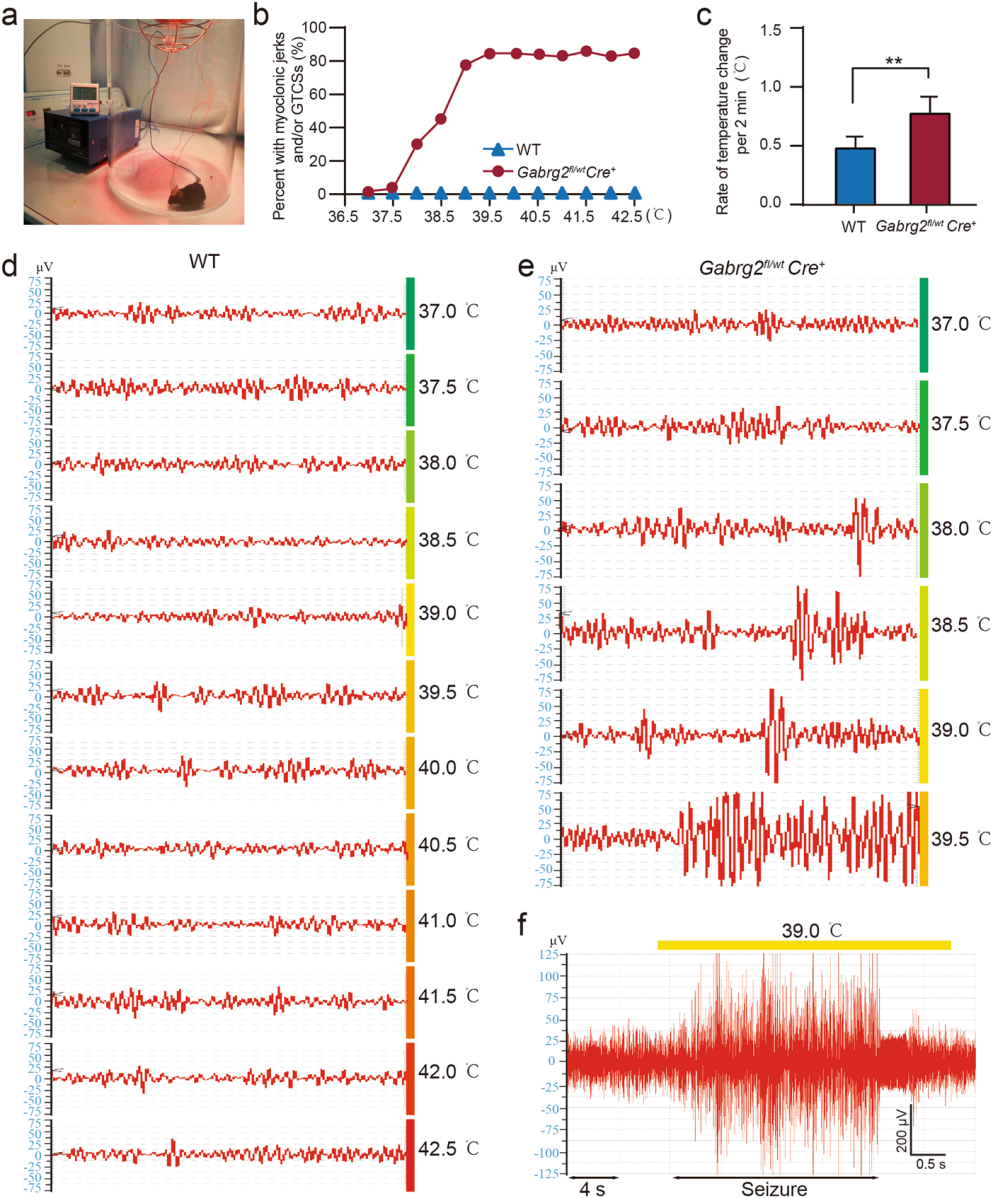
Seizures induced by temperature rising and abnormal electroencephalography (EEG) activity in *Gabrg2*^*fl/wt*^*Cre*^*+*^ conditional knockout mice. **a** Photograph of the temperature controller and heating setup. **b** Percentage of myoclonic jerks and/or generalised tonic-clonic seizures (GTCS) was plotted for the adult *Gabrg2*^*fl/wt*^*Cre*^*+*^ and WT mice with rising body temperature. For adult *Gabrg2*^*fl/wt*^*Cre*^*+*^, more than 80% of mice have temperature-induced seizures at 39.5 °C; however, WT mice did not. **c** The rate of core body temperature changes in adult *Gabrg2*^*fl/wt*^*Cre*^*+*^ and WT mice was measured every 2 min during the heating process. The change rate of core temperature per 2 min was higher in *Gabrg2*^*fl/wt*^*Cre*^*+*^ (*n* = 21) than in WT (*n* = 24) mice (*P* < 0.01). **d** Representative 1 s traces of intracranial EEG activity for WT mice during temperature elevation until 42.5 °C. There was no seizure above stage 3 observed in any WT mouse during heating process (*n* = 24). **e** Representative intracranial EEG traces of *Gabrg2*^*fl/wt*^*Cre*^*+*^ mice during temperature-induced seizures. With the temperature rising, the higher spikes and amplitudes were seen. **f** The GTCSs was induced at 39 °C. Data shown are mean ± standard error of mean. **P < 0.01 vs WT, *t*-test (two-tailed).

Fever plays a critical role in the occurrence of GEFS +, which occurred in early childhood (3 months to 6 years)^1,38^. We used a temperature heating controller on *Gabrg2*^*fl/wt*^*Cre*^*+*^ and WT mice (Fig. 6a). The baseline core temperatures of all mice were ~36.9 °C, and the temperatures of *Gabrg2*^*fl/wt*^*Cre*^*+*^ (*n* = 21) and WT (*n* = 24) mice were 36.81 ± 0.47 and 36.73 ± 0.51 °C, respectively. The heating protocol was used as previously described^37,39^, which stopped until the animal’s core body temperature reached 42.5 °C or until GTCS occurred. More than 80% of mice (*n* = 17/21) had MJs and GTCSs during temperature elevation. WT mice had no MJs and/or GTCSs, even at 42.5 °C. Rate of MJs and/or GTCSs did not increase with increasing temperature when the core body temperature reached 39.5 °C in *Gabrg2*^*fl/wt*^*Cre*^*+*^ mice (Fig. 6b). The first myoclonic seizure and/or GTCSs were recorded at 38 °C in *Gabrg2*^*fl/wt*^*Cre*^*+*^ mice (Video 2) during heating, more than 50% of the heterozygotes had seizures at 39 °C, and the temperature-induced seizures occurred at an average temperature of 38.7 ± 1.22 °C (*n* = 21). The rate of temperature change per 2 min was used as an indication for temperature sensitivity. There was a faster rate of temperature change in *Gabrg2*^*fl/wt*^*Cre*^*+*^ mice than in their WT controls (*P* = 0.0034) (Fig. 6c). The core body temperature of mice was increased by 0.5 °C per 2 min, simulating a fever process. There were no MJs or GTCSs recorded in WT mice (Fig. 6d) during heating, whereas most of the *Gabrg2*^*fl/wt*^*Cre*^*+*^ mice had the first SWDs or even GTCSs at 38 °C (Fig. 6e). Temperature-induced seizures in *Gabrg2*^*fl/wt*^*Cre*^*+*^ mice had a short latent period, and the mice returned to normal state, with normal EEG, once seizures ended (Fig. 6f). These data suggest that temperature rise alone is sufficient to reliably induce seizures in *Gabrg2*^*fl/wt*^*Cre*^*+*^ mice.

### Pentylenetetrazole (PTZ)-induced seizures in *Gabrg2*^*fl/wt*^*Cre*^*+*^ mice

**Fig. 7:**
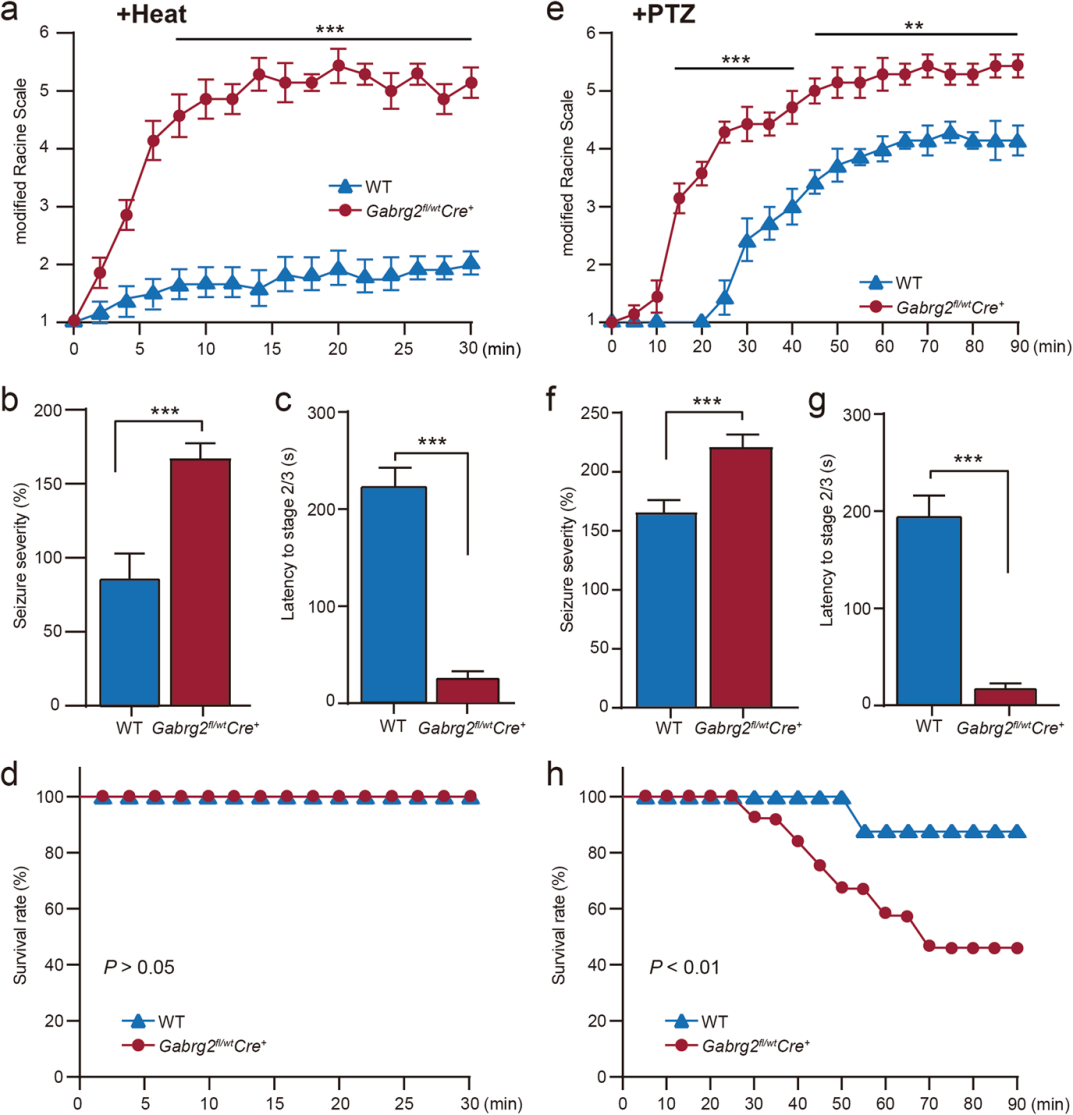
Temperature- and pentylenetetrazole (PTZ)-induced seizure latency, severity, as well as survival between *Gabrg2*^*fl/wt*^*Cre*^*+*^ and wild type mice. **a** The modified Racine grade was used to evaluate the scores of seizure severity between *Gabrg2*^*fl/wt*^*Cre*^*+*^ (*n* = 21) and WT (*n* = 24) mice during the heating process. The severity of seizures increased markedly in *Gabrg2*^*fl/wt*^*Cre*^*+*^ mice compared to WT mice. **b** A value of 100% was allocated to the average seizure severity score of the WT mice, and then applied to normalise the severity of the *Gabrg2*^*fl/wt*^*Cre*^*+*^ mice within the same scale. The seizure severity was significantly higher in *Gabrg2*^*fl/wt*^*Cre*^*+*^ mice compared to WT mice during temperature-induced seizure. **c** The latency to reach stages 2/3 of the modified Racine scale was significantly different between *Gabrg2*^*fl/wt*^*Cre*^*+*^ and WT mice for temperature-induced seizures. **d** Survival percentage indicated that there was no significant difference (0 died for WT vs. 0 died for *Gabrg2*^*fl/wt*^*Cre*^*+*^, *P* > 0.05) in survival between *Gabrg2*^*fl/wt*^*Cre*^*+*^ and WT mice over 30 min during temperature-induced seizure process. **e** The modifi**e**d Racine grade was used to evaluate the scores of seizure severity between *Gabrg2*^*fl/wt*^*Cre*^*+*^ (*n* = 21) and WT (*n* = 24) mice during PTZ-induced seizures. The severity of seizure increased significantly in *Gabrg2*^*fl/wt*^*Cre*^*+*^ mice as compared with WT mice. **f** The seizure severity was significantly higher in *Gabrg2*^*fl/wt*^*Cre*^*+*^ mice compared with WT mice for PTZ- induced seizure. **g** The latency to reach stages 2/3 of the modified Racine scale was significantly different between *Gabrg2*^*fl/wt*^*Cre*^*+*^ and WT mice for PTZ-induced seizures. **h** The mortality increases significantly in *Gabrg2*^*fl/wt*^*Cre*^*+*^ mice compared with WT mice after intraperitoneal injection of PTZ (11/21 died for *Gabrg2*^*fl/wt*^*Cre*^*+*^ vs. 1/24 died for WT). Data shown are mean ± standard error of mean. **P < 0.01 and ***P < 0.001 vs WT, *t*-test (two-tailed).

The modified Racine Scale was used to score the severity of PTZ- or elevated temperature-induced seizures in WT and *Gabrg2*^*fl/wt*^*Cre*^*+*^ mice. The WT mice had no seizures (below stage 2) during heating. The *Gabrg2*^*fl/wt*^*Cre*^*+*^ mice experienced significantly more severe seizures (above stage 4) than WT mice with temperature induction (Fig. 7a and b). The severity of seizure was used to evaluate the seizure differences between the two groups, as in our previous study^40^. The average latency to reach stage 2 or 3 of the modified Racine Scale in *Gabrg2*^*fl/wt*^*Cre*^*+*^ mice (*n* = 21) was significantly shorter than that in WT mice (*n* = 24) during temperature-induced seizures (*P* < 0.001) (Fig. 7c). All mice had anxiety-like symptoms (jumping bouts) during the heating process, especially at 38.5–39.5 °C (Video 3), which are not only associated with anxiety-related psychopathological conditions but also are a behavioural anxiety marker in addition to seizure-related activity^37,41^. The number of jumping frequencies was higher in *Gabrg2*^*fl/wt*^*Cre*^*+*^ mice than in WT mice during the heating process (*P* < 0.001) (Fig. S4a). Moreover, the average temperature of the initial jumping of *Gabrg2*^*fl/wt*^*Cre*^*+*^ mice was lower than that of WT mice (*P* < 0.01) (Fig. S4b). We collected mortality data from the WT and *Gabrg2*^*fl/wt*^*Cre*^*+*^ mice experiencing temperature-induced seizures during the heating process. There was no death in WT or *Gabrg2*^*fl/wt*^*Cre*^*+*^ mice during temperature elevation (*n* = 0/21 for *Gabrg2*^*fl/wt*^*Cre*^*+*^ vs. *n* = 0/24 for WT mice, Kaplan–Meier test; *P* > 0.05) (Fig. 7d).

The modified Racine grade scores at 5 min intervals after PTZ intraperitoneal injection and the evaluation of seizure severity between the *Gabrg2*^*fl/wt*^*Cre*^*+*^ and WT mice groups were recorded. The results indicated that all *Gabrg2*^*fl/wt*^*Cre*^*+*^ mice had seizures of above stage 4 in a short time as compared with WT mice; only few WT mice reached stage 4 and/or above stage 5 (Fig. 7e and f). The *Gabrg2*^*fl/wt*^*Cre*^*+*^ mice experienced significantly more severe seizures (above stage 4) than WT mice (*P* < 0.001) (Fig. 7g). More *Gabrg2*^*fl/wt*^*Cre*^*+*^ mice died (*n* = 11/21) than WT mice (*n* = 1/24) following PTZ-induced seizures (Kaplan–Meier test; *P* < 0.01) (Fig. 7h). These results indicate that high temperature-induced seizures were less severe than PTZ-induced seizures.

### *Gabrg2*^*fl/wt*^*Cre*^*+*^ mice displayed anxiety-like behaviours

**Fig. 8:**
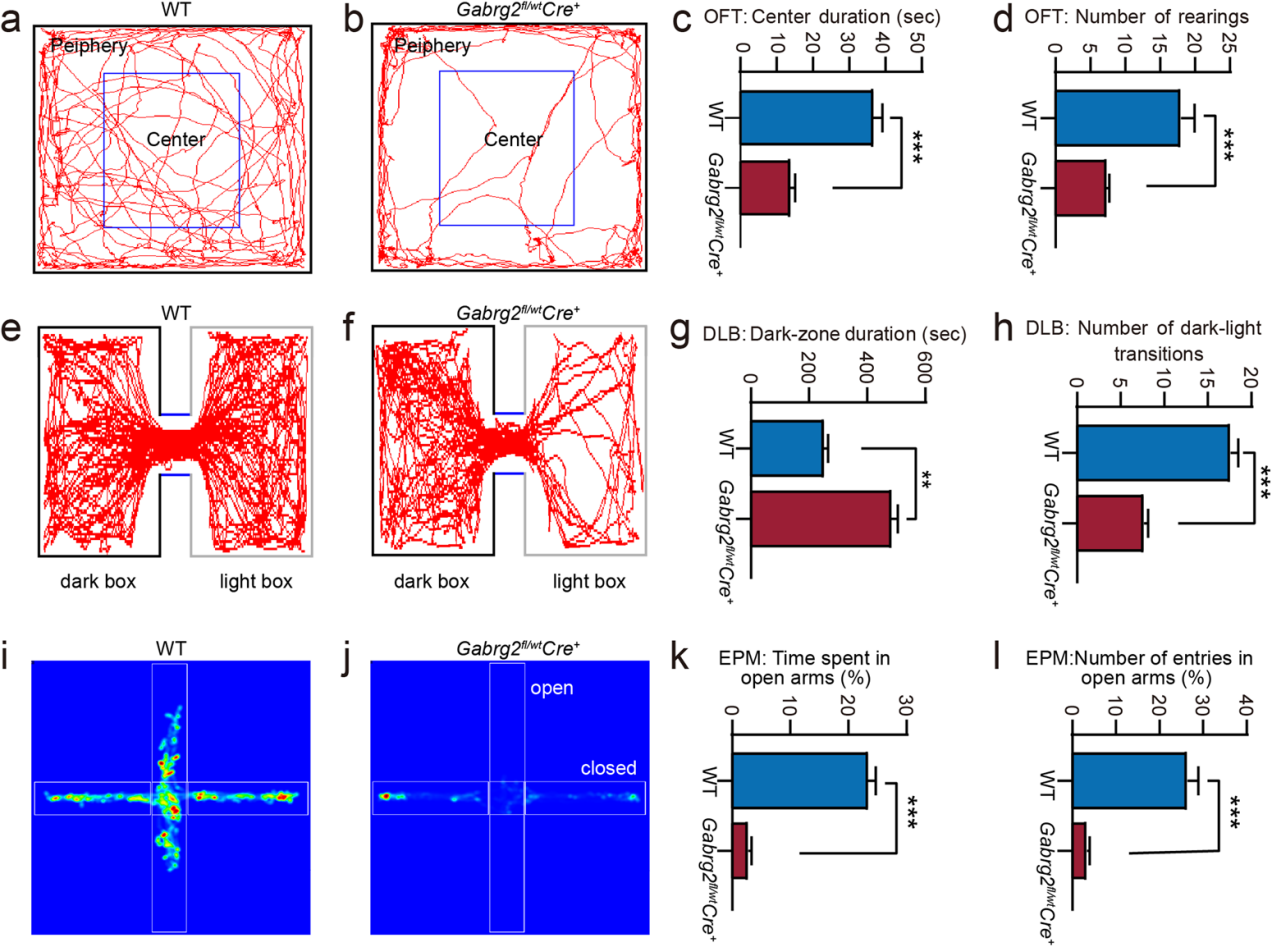
*Gabrg2*^*fl/wt*^*Cre*^*+*^ mice display increased anxiety-like behaviours by open field (OF), dark-light box (DLB), and elevated-plus maze (EPM) tests. **a**, **b** Representative trace of a mouse’s movements in each group in the OF test. **c** *Gabrg2*^*fl/wt*^*Cre*^*+*^ mice spent significantly less time in the central area of the field (14.20 ± 6.34 s for *Gabrg2*^*fl/wt*^*Cre*^*+*^ mice and 37.11 ± 10.75 s for WT, *P* < 0.001) and **d** had decreased number of rearing (7.40 ± 2.67 for *Gabrg2*^*fl/wt*^*Cre*^*+*^ mice and 18.00 ± 7.51 for WT, *P* < 0.001) than WT mice (*n* = 12 for WT and *n* = 12 for *Gabrg2*^*fl/wt*^*Cre*^*+*^ mice). **e**, **f** Representative trac**e** of a mouse’s movements in each group in DLB test. **g** Compared with the WT **g**roup, *Gabrg2*^*fl/wt*^*Cre*^*+*^ mice had a significant increase in the time spent in the dark zone (252.87 ± 81.93 s for WT and 485.41 ± 109.77 s for *Gabrg2*^*fl/wt*^*Cre*^*+*^ mice, *P* < 0.001) and **h** a decrease in the number of DLB entries (17.60 ± 3.48 for WT and 7.67 ± 1.84 for *Gabrg2*^*fl/wt*^*Cre*^*+*^ mice, *P* < 0.001) (n = 12 per group). **i**, **j** Representative heat map analysis of a mouse in each group. **k** The percentage of open arms time (%) and **l** percentage of open arms entries (%) in the EPM test in WT and *Gabrg2*^*fl/wt*^*Cre*^*+*^ mice (*n* = 12 for each genotype). Data shown are mean ± standard error of mean. **P < 0.01 and ***P < 0.001 vs WT, *t*-test (two-tailed).

We performed several tests to assess the behaviour phenotype of *Gabrg2*^*fl/wt*^*Cre*^*+*^ mice. The open field test (OFT) results showed that the total travelling distance was significantly increased in the *Gabrg2*^*fl/wt*^*Cre*^*+*^ mice compared with WT mice (*n* = 12 for each group) (40.27 ± 7.19 m for *Gabrg2*^*fl/wt*^*Cre*^*+*^ and 10.14 ± 3.21 m for WT; *t*-test, *P* < 0.001). The computer-generated traces of the animal’s movements were shown in Fig. 8a and b. *Gabrg2*^*fl/wt*^*Cre*^*+*^ mice spent significantly less time in the central area of the field (*P* < 0.001) than WT mice and had decreased number of rearing (*P* < 0.001) (Fig. 8c and d).

To assess the anxiety-like behaviour in *Gabrg2*^*fl/wt*^*Cre*^*+*^ mice, we used the dark-light box (DLB) test. The increased time spent in the brightly lit side of the apparatus serves as an indicator of less anxiety-like behaviour. The DLB results showed the changes in the latency to enter the light compartment and the number of DLB entries in *Gabrg2*^*fl/wt*^*Cre*^*+*^ mice. The traces of the animal’s movements were shown in Fig. 8e and f. Compared with the WT mice (*n* = 12), *Gabrg2*^*fl/wt*^*Cre*^*+*^ mice (*n* = 12) had a significant increase in the time spent in the dark zone (*P* < 0.01) and a decrease in the number of DLB entries (*P* < 0.001) (Fig. 8g and h).

We further performed the elevated-plus maze test (EPM). This test depends on the assumption that mice inherently prefer the closed arms of the maze to the open arms. The representative heat maps of one animal from each group were shown in Fig. 8i and j. The time spent on the open arm of the EPM was expressed as a percentage of the total time spent on any arm during the 5-min test. The *Gabrg2*^*fl/wt*^*Cre*^*+*^ mice (*n* = 12) appeared to spend less time on the open arms (23.47 ± 6.50% for WT and 2.76 ± 2.11% for *Gabrg2*^*fl/wt*^*Cre*^*+*^ mice, *P* < 0.001) (Fig. 8k), whereas WT mice (*n* = 12) exhibited a higher proportion of entries into the open arms (26.12 ± 4.22% for WT and 3.17 ± 1.26% for *Gabrg2*^*fl/wt*^*Cre*^*+*^ mice, *P* < 0.001) (Fig. 8l). These results suggest that *Gabrg2* deletion in hippocampus and neocortex affects anxiety-like responses.

## Discussion

We created a typical mouse model of temperature-induced FS or GEFS + by targeted deletion of *Gabrg2* in neurons of hippocampus and neocortex (heterozygous *Gabrg2*^*fl/wt*^*Cre*^*+*^ mice). Increasing the core body temperature in *Gabrg2*^*fl/wt*^*Cre*^*+*^ mice induced ictal discharges, resulting in a significant increase in GTCS events. This supports previous studies in *Gabrg2*^*+/R43Q*^ and *Gabrg2*^*+/Q390X*^ mice that mutant γ2 (R43Q) and γ2 (Q390X) subunits increase susceptibility to FS or GTCS during core body temperature elevation^37,42^. Constitutive *Gabrg2*^+/-^ knockout mice were first generated by Günther^36^, and the mice only exhibited the absence seizure phenotype^43^. In 2007, *Gabrg2* (R43Q) knockin mouse model was successfully constructed, and the mice displayed an absence epilepsy, GEFS + , and febrile seizure^44,45^. The intronic *Gabrg2* mutation (*IVS6* + *2* *T* → *G*) that was established by Tian^46^ could induce absence epilepsy by both γ2 subunit haploinsufficiency and γ2-PTC subunit dominant-negative functions. Moreover, a heterozygous *Gabrg2*^*+/Q390X*^ knockin mouse that was generated by Kang^24^, displayed a variety of phenotypes, such as FS, FS + , GTCSs, GEFS + , Dravet syndrome, or even sudden unexpected death in epilepsy^24,37,46^. We have summarised various *GABRG2* mutations-associated epilepsy phenotypes in humans and animal models in Supplementary Table S3.

The deletion of *Gabrg2* (homozygous) in the neocortex and hippocampus was incompatible with brain function and resulted in growth delay, and ultimately death perinatally with rare survivors reaching postnatal day 21, while heterozygous *Gabrg2*^*fl/wt*^*Cre*^*+*^ mice were normally viable. We did not observe excessive hyperactivity in homozygous mice, which is different from previous studies^35,36^. Our Nissl staining revealed the marked loss of neurons in the hippocampus and cortex layers V–VI in *Gabrg2*^*fl/wt*^*Cre*^*+*^ mice. This suggests that the γ2 subunit may play an important role in neuronal apoptosis in *Gabrg2*^*fl/wt*^*Cre*^*+*^ mice. Indeed, it has been shown that γ2 subunit-contaning GABA_A_R is essential for cortical interneuron apoptosis^47^.

*Gabrg2* ^*fl/wt*^*Cre*^*+*^ mice experienced their first spontaneous seizures in the fourth postnatal week, and persisted at 2–4 months of age, indicating that *Gabrg2* plays an important role in epileptogenesis. The mouse phenotype resembles GEFS + in humans in its severity, early age of onset, and striking dependence on genetic background^8,9,13,15,37,48^. The physiological mechanisms underlying reduced seizure threshold with elevated body temperature are not well understood. As the γ2 subunit is critical for GABA_A_R clustering^34,49^, trafficking^50^, and synaptic maintenance^51^, it is reasonable to consider that defecient γ2 subunit contributes to the temperature-dependent seizures in this mouse model.

Temperature-induced seizures were less severe than PTZ-induced seizures in *Gabrg2*^*fl/wt*^*Cre*^*+*^ mice. Core temperature elevation and PTZ injection were effective in producing the SWD characteristics of epilepsy, but the effect of heating was not as strong as that of PTZ administration. These findings suggest that both induction approaches may reduce the inhibitory tone in the brain. During the heating process, especially between 38.5 °C and 39.5 °C, an anxiety-like behaviour occurred in *Gabrg2*^*fl/wt*^*Cre*^*+*^ mice. Moreove, a previous study has showed that *Gabrg2*^+/Q390X^ knockin mice have a similar anxiety-like behaviour using the jumping rate as an indicator of anxiety events^37^. These results are consistent with previous findings in heterogenous *Gabrg2*^+/-^ constitutive knockout mice^52^. The γ2 subunits are broadly expressed in brain regions related to anxiety, including the forebrain, hippocampus, and amygdala^52^. It has been shown that anxiety is deemed as a sign of impaired GABAergic neurotransmission^53^. Dysfunctions in the GABAergic system, particularly genetic mutations of GABA_A_R, have been known to evoke anxiety^33,52,54,55^. In the current study, anxiety-like behaviours were evaluated using the OF, DLB, and EPM tests. Several indices, including decreased percentage of time spent in the central zone and the number of rearing in the OF test, lower time spent in the light box and the number of DLB transitions, and a lower percentage of time spent in open arms and the frequency of open arm entries in the EPM, indicate the presence of anxious behaviours in adult *Gabrg2*^*fl/wt*^*Cre*^*+*^ mice. The anxiety phenotype displayed in *Gabrg2*^*fl/wt*^*Cre*^*+*^ mice supports the notion that the GABA_A_R deficiency is a predisposition for anxiety disorders at the clinical level.

We have observed the higher rate of temperature change per 2 min in adult *Gabrg2*^*fl/wt*^*Cre*^*+*^ mice compared with WT mice under the same heating parameters. The preoptic anterior hypothalamus is the principal centre for body temperature regulation. Although the expression of the γ2 subunit in hypothalamus neurons may not be affected in *Gabrg2*^*fl/wt*^*Cre*^*+*^ mice, the hypothalamus activity can be modulated via direct or indirect neural projections from neurons in the hippocampus and neocortex^56^. The underlying mechanism involved in this alteration is unknown and needs further investigation.

In humans, long-term and complex FS/FS+ promotes the late development of mesial temporal epilesy associated with focal cortical dysplasia and hippocampal sclerosis^57^, which indicates that prolonged complex FS/FS+ may induce neuronal injury. Our previous study has shown that *Gabrg2* dysfunction affects the expression of other subunits of the GABA_A_R and voltage-gated calcium channel subunit (Cacna1a) in vitro, which may contribute the epileptic seizure^58^. However, the effect of brief exposure to increased temperature has not been addressed. It is likely that the epileptic syndrome in *Gabrg2*^*fl/wt*^*Cre*^*+*^ mice has the similar cellular and molecular basis as GEFS+ in humans, which would provide a unique model for the investigation of this disease.

We introduce a new in vivo genetic model of idiopathic epilepsy that is convenient for a broad range of scientific applications. The FS/FS + phenotype can be induced by heating stimuli greatly improves the standardisation of the seizure assays, thus potentially facilitating further drug-screening applications.

## Materials and methods

### Animals

Female and male C57BL/6 J and B6.129S2-Emx1^tm1(cre)Krj^/J (Emx1-IRES-Cre, Stock No.: 0056289) mice were purchased from the Experimental Animal Centre of Ningxia Medical University and the Jackson Laboratory, respectively. Mice aged 8–18 weeks were housed according to specific pathogen-free grade animal feeding standards at 22–28 °C indoor, 40–60% humidity, and a 12 h light/dark cycle (lights on 7 am; lights off 7 pm) in an individual cage ventilation system. The mice were fed a standard diet after sterilisation and allowed access to water and food *ad libitum*. All experimental procedures were reviewed and approved by the Institutional Animal Care and Use Committee of Ningxia Medical University [IACUC Animal Use Certificate No.: SCXK (Ning) 2019-203].

### CRISPR/Cas9 system and generation of *Gabrg2*-floxed mice

*Gabrg2*-floxed mice (*Gabrg2*^*fl/wt*^) were generated using CRISPR/Cas9-mediated genome engineering techniques. We inserted two *loxP* sites flanking exon 2 of the *Gabrg2* gene transcript (ENSMUST00000070735.9) with the 5’-ATAACTTCGTATAATGTATGCTATACGAAGTTAT-3’ and 5’-ATAACTTCGTATAATGTATGCTATACGAAGTTAT-3’ sites inserted in the upstream and downstream intronic sequences, respectively. First, two sgRNAs targeting the introns on both sides of the *Gabrg2* floxed region were constructed and transcribed in vitro. The donor vector with the *loxP* fragment was designed and constructed in vitro. Thereafter, Cas9 mRNA, sgRNA, and donor were co-injected into zygotes. The zygotes were transferred into the oviduct of pseudo pregnant ICR females at 0.5 dpc. F_0_ mice were born 19–21 days after transplantation, and all offspring of ICR females (F_0_ mice) were identified by PCR and sequencing of tail DNA. Positive F_0_ mice were genotyped by the same methods. Finally, F_0_ mice were crossed with C57BL/6 J mice to produce heterozygous mice (genotype: fl/wt; F_1_).

### DNA extraction, PCR, and sequencing

After blastocyst transfer, the new-born mice were genotyped by PCR and sequencing using their genomic DNA from toes or tail tissues. To detect whether the *loxP* site and other sites were correctly inserted, we designed specific primers to confirm the genotype of mice. The primer sequences are shown in Supplementary Table S1. First, we designed primers (primer No. 1) across both ends of a *loxP* site to detect the wild type and tested whether the *loxP* site was correctly inserted. The 3’ preliminary screening probe (primer No. 2) was used for reverse testing. Second, we tested whether the *loxP* of the 5’ ss-DNA and 5’ homologous arm was correct using primer No. 3. Third, the *loxP* of the 3’ ss-DNA and 3’ homologous arm were also tested with primer No. 4. Last, we confirmed whether the *loxP* of the 5′ and 3’ sites were in the same allele using primers No. 5 and No.6. Mice No. 40, 41, 45, 49, and 50 were deemed chimeric mice with the genotype *Gabrg2*^*fl/wt*^. The PCR products of mice No. 40, 41, 45, and 49 were sequenced (mouse No. 50 died of weakness). For each sample, the reaction was performed in a mixture (25 µL) containing 1.25 µL forward primer and reverse primer, 12.5 µL Q5 Hot Start High-Fidelity DNA polymerase (#M0494X; New England Biolabs, Ipswich, MA, USA), and 500 ng DNA. F_0_ and F_1_ mice were tested according to the different primer and reagent sets. The PCR parameters were set as follows: initial denaturation at 98 °C for 30 s, followed by 35 cycles of denaturation at 98 °C for 10 s, annealing at 50–72 °C for 30 s, extension at 72 °C for 30 s, and final extension at 72 °C for 2 min (Gene Amp^®^ PCR System 9700; Applied Biosystems, Foster City, CA, USA).

### Generation and genotyping of the neocortex- and hippocampus-specific CKO mouse

*Gabrg2* CKO mice were generated by crossing *Gabrg2*^*fl/wt*^ mice with Emx1-IRES-Cre homozygous mice. Genomic DNA was isolated from toes or tails using a TIANamp Genomic DNA Kit (#DP 304; Tiangen Biotech, Beijing, China) according to the manufacturer’s protocol. The DNA concentration was determined using a spectrophotometer (Nanodrop 2000; Thermo Fisher Scientific, Waltham, MA, USA). DNA deletion was identified by standard PCR using DNA polymerase and specific primers, which are listed in Supplementary Table S2. PCR products were analysed by agarose gel electrophoresis. The majority of functional investigations were carried out on 2- to 4-month-old heterozygous mice (*Gabrg2*^*fl/wt*^*Cre*^*+*^) from the F_3_ generation. Few old animals (6–8 months) were also included for comparison. To avoid potential carryover effects, all animals were used only once throughout the study.

### RNA isolation and RT-qPCR

Total RNA was extracted from the mouse cortex and hippocampus as previously described^40^. First-strand cDNA was generated using 5 × All-In-One RT Master Mix reverse transcriptase (#G486; Applied Biological Materials Inc., Richmond, Canada) and used as the qPCR template. The cDNA was amplified using a one-step qPCR kit (#Master Mix-ES; Applied Biological Materials Inc.). The *Gabrg2* primers were (F) 5-TAGCACGGCTTGATTCTTGC-3 and (R) 5-TGCATTCCATGCTGTT

CTCC-3, and the *Gapdh* primers were (F) 5-GAGTCAACGGATTTGGTCGT-3 and (R) 5-GACAAGCTTCCCGTTCTCAG-3′. The qPCR parameters were as follows: an initial denaturation at 95 °C for 30 s, followed by 40 cycles of denaturation at 95 °C for 5 s, annealing/extension at 60 °C for 30 s (Bio Rad^®^ CFX96; Bio-Rad Laboratories, Hercules, CA, USA). After the amplification, Bio-Rad IQ5 software was used for data analysis, and *Gapdh* was used as an internal reference. The comparative threshold (2^-∆∆Ct^) method was used, and the results were converted to fold change relative to the WT group.

### Western blot

Animals were anaesthetised with isoflurane (#R510-22; RWD Life Science Co., Ltd, Shenzhen, China) and euthanised by decapitation. The hippocampus, cortex, and other brain regions were dissected on ice and subsequently homogenised using a Vibra-Cell™ Ultrasonic Liquid Processors (#VCX 130PB; Sonics & Materials Inc., Newtown, CT, USA) in lysis buffer consisting of phosphatase and protease inhibitors and phenyl methane sulphonyl fluoride. After centrifuging for 10 min at 12,500 *g* (4 °C), the protein concentrations were determined. Before boiling at 100 °C for 5 min to denature, 80 μg of protein sample was added to the loading buffer. Protein signals were visualised using the Odyssey CLx imaging system (#9141-00; LI-COR Biosciences, Lincoln, NE, USA) according to the manufacturer’s instructions. GAPDH was used as the internal reference. Representative immunoblotting results from at least three independent experiments were used for analysis. Antibodies used in western blotting were as follows: rabbit polyclonal anti-GABRG2 (ab87328; 1:1 000; Abcam, Cambridge, MA, UK) and mouse monoclonal anti-GAPDH (#60004-1-Ig; 1:5 000; Proteintech Group, Inc., Wuhan, China); secondary antibodies were IRDye^®^800RD Goat anti-Mouse IgG (H + L) (#926-32210; 1:5 000; LI-COR Biosciences) and IRDye^®^680RD Goat anti-Rabbit IgG (H + L) (#925-68071).

### Immunohistochemistry

Under isoflurane anaesthesia, the mice were transcardially perfused with 0.9% physiological saline (50 mL), followed by 4% paraformaldehyde (50–60 mL). Brains were quickly removed and postfixed in 4% paraformaldehyde (#30525-89-4; Sigma-Aldrich Inc., St. Louis, MO, USA) at 4 °C for 12 h, followed by 15%, 20% and 30% sucrose gradient dehydration for a day, until the brain completely sink to the bottom. The brains were embedded using the optimal cutting temperature compound, and then treated briefly with liquid nitrogen. The brain tissues were cut on a cryostat (CM1950; Leica, Wetzlar, Germany), and sections (15 µm in thickness) were collected. Briefly, sections were rinsed in phosphate-buffered saline (PBS) and treated with 3% H_2_O_2_ for 10 min to inactivate endogenous peroxidase activity. Sections were transferred to 0.01 M pH 6.0 citric acid at 100 °C for 15 min and cooled to room temperature (RT), followed by PBS 3 × 3 min. The sections were then incubated with 5% bovine serum albumin for 1 h at 37 °C to reduce nonspecific staining. After rinsing in PBS for 3 min (3 times), sections were incubated with rabbit anti-GABRG2 antibody (#PA5-77404; 1:200; Invitrogen, Waltham, MA, USA) overnight at 4 °C. Following 3 min (3 times) PBS washes, sections were incubated with biotinylated goat anti-rabbit IgG (#PV-6001; 1:500; ZSGB-Bio, Beijing, China) for 1 h at RT. After rinsing with PBS for 3 min (3 times), the setions were incubated with 5% 3,3’-diaminobenzidine (DAB) (#ZLI-9018; ZSGB-Bio, Beijing, China) for 5–10 min at RT. The sections were further counterstained with haematoxylin for 1–2 min at RT, dehydrated with gradient alcohol (70% for 3 min, 80% for 3 min, 95% for 3 min, 100% I for 2 min, 100% II for 2 min), cleaned in xylene I for 5 min, and xylene II for 5 min. The samples were sealed with neutral balsam (#G8590; Solarbio, Beijing, China) on slides and observed under a light microscope (DM6; Leica, Wetzlar, Germany). The number of Gabrg2 positive cells was counted per high-power field (×400) in every two random regions of interest, and Image-Pro Plus 6.0 software (Media Cybernetics, Inc., Rockville, MD, USA) was used to analyse intergroup differences.

### Nissl staining

Tissue sections were prepared as described in immunohistochemistry. The cresyl violet solution (#G1430; Solarbio, Beijing, China) was used to stain brain sections at 56 °C for 60 min, and then rinsed briefly three times with deionised water. The samples were incubated in Nissl differentiation solution for a few seconds to 2 min, then quickly followed by dehydration with gradient alcohol as described above, cleaned with xylene I for 5 min and xylene II for 5 min, and mounted with neutral balsam. We counted and analysed the cells of Nissl staining from different groups.

### Electrode implantation, video/EEG monitoring, recording and analysis

Under isoflurane (3% for induction and 2% for maintenance) anaesthesia, 2- to 4-month-old (22–28 g body weight) mice underwent surgery for implantation of EEG electrodes. To enhance the stability of electrodes and reduce the injury of dental cement to the skin and eyes around the incision, we designed a novel apparatus. In brief, a midline incision of the scalp was made after successful anaesthesia in mice, and the prepared electrodes were implanted into the drilled holes. Video/EEG monitoring and recording were carried out as described in the Supplementary Information Text.

### Seizures induction by temperature rise

The heating protocol was performed as previously described^37^. The rectal temperature probe (#RET-4; Physitemp Instruments Inc., Clifton, NJ, USA) was carefully inserted into the anus of mice and taped to the tail, and then connected to a rodent temperature controller (#TCAT-2DF; Physitemp Instruments Inc.). Mice were placed in a Plexiglas cylinder (diameter: 50 cm; height: 30 cm; thickness: 5 mm) with an infrared heat lamp (#HL-1; Physitemp Instruments Inc.) kept in a fixed position. The average body core temperature of mouse is 36.9 °C (http://www.informatics.jax.org/mgihome/other/mouse_facts1.shtm), and the core body temperature was monitored by the temperature controller. Each mouse was held in a Plexiglas cylinder without the heat lamp light for at least 10 min to acclimate to the chamber. The body temperature was recorded for 30 min to 2 hours under baseline activity. The heat lamp was turned on, and the EEG recording began before temperature elevation; the temperature was elevated about 0.5 °C every 2 min until a seizure occurred or until 42.5 °C was achieved. The rate of temperature rise in mice was determined by the height of the heating lamp. Once the core temperature reached 42.5 °C or a GTCS event occurred, the heating process would be stopped immediately.

### PTZ-induced seizures

PTZ is a GABA_A_R antagonist that induces generalised tonic-clonic seizures after intraperitoneal injection. Seizures were induced using PTZ as previously described^37,59–61^. In the present study, adult mice were intraperitoneally injected with a single dose of PTZ (30 mg/kg) (#P6500; Sigma-Aldrich Inc.) dissolved in 0.9% saline, and recorded during the first 30 min after administration. GTCSs were terminated by diazepam administration (1 mg/kg).

### Behavioural tests

All behavioural tests were performed in a quiet and brightly lit room between 08:00 and 17:00. The animals were transported to the experimental room 30 min prior to testing (habituation period). Behavioural activities were recorded and subsequently analysed by an experimenter blinded to the animal groups. To investigate anxiety-like behaviours in 8–18-week-old mice, OF, DLB, and EPM tests were performed. All animal behaviours were recorded and analysed for 5 or 10 min using the SMART video tracking system (SMART 3.0, Panlab SL Inc., Barcelona, Spain, supported by RWD Life Science Co., Ltd).

*OF test*. The open-field apparatus was constructed using a polyvinyl chloride chamber (45 × 45, 40 cm height) and a white floor divided into 20 squares of equal size. Individual mouse was placed in the centre of the square arena and allowed to freely explore the arena for 5 min. Time in the central/marginal zone and the number of rearing were recorded and analysed during the test period. The device was cleaned between each individual mouse test session using 75% ethanol and dried.

*DLB test*. The dark-light experimental box was made of Perspex and composed of a small black compartment (16 × 25 × 24 cm) and a big white compartment (25 × 25 × 24 cm) separated by a connecting gate (7 × 7 cm). The light chamber was painted white, and the dark chamber was painted black. Animals were placed in the light compartment, the time spent in the light/dark zones and number of zone transitions were recorded as indictors of anxiety during a 10 min trial. A light box visit was recorded when the mouse moved at least half of its body into the light box.

*EPM test*. The apparatus consisted of a plus-shaped grey polyvinyl chloride maze with two open arms without side walls (30 × 6 × 1.8 cm), two arms closed by side walls (30 × 6 ×15 cm), and a central platform (6 × 6 cm), which was elevated to a height of 50 cm. To start testing, mice were individually placed on the central maze, with the head toward a closed arm and allowed to explore it for 5 min. After each trial, the apparatus was cleaned with 75% ethanol. The duration of visits in both the open and closed arms was recorded. The percentage of entries and time spent in open arms (open arms/[open arms + closed arms] × 100) were analysed to minimise the bias due to possible differences in locomotor activity. An entry into an open arm was defined as all four paws crossing the centre of the maze.

### Statistical analysis

GraphPad Prism 8 (GraphPad Software Inc., San Diego, CA, USA) and the statistical package for the social sciences (SPSS) 23.0 software (IBM Inc., Armonk, NY, USA) were used for statistical analysis. All data were presented as mean ± standard error of the mean. The assessment of data normality was performed with Kolmogorov-Smirnov normality test. Data with normal distributions were analysed by Student’s *t*-test, whereas data with non-normal distributions were analysed by Mann–Whitney test when comparing two groups. The Gabrg2 mRNA/protein levels and body weight among different groups were analysed using one-way ANOVA with Tukey’s multiple comparisons *post hoc* test. Survival rate was assessed using the Kaplan–Meier test. Unless otherwise specified, *n* represented the number of animals used. All analyses used α = 0.05 to evaluate statistical significance.

## Supplementary information

Supplementary

SI Fig.1

SI Fig.2

SI Fig.3

SI Fig.4

Video 1

Video 2

Video 3
