# Supplementary material for: Neocortex- and hippocampus-specific deletion of Gabrg2 causes temperature-dependent seizures in mice
Source: Cell Death Dis. 2021 May 28;12(6):553. doi: 10.1038/s41419-021-03846-x (PMC8163876; doi:10.1038/s41419-021-03846-x)
Supplement: Supplementary file 1 — Supplementary [file 41419_2021_3846_MOESM1_ESM.docx]

**Cell death and Disease**

Supporting information for

**Neocortex- and hippocampus-specific deletion of *Gabrg2* causes temperature-dependent seizures in mice**

Xinxiao Li, Shengnan Guo, Siying Xu, Zhangping Chen, Lei Wang, Jiangwei Ding, Junming Huo, Lifei Xiao, Zhenquan He, Zhe Jin, Feng Wang, Tao Sun

Correspondence to

Tao Sun, M.D., Ph.D., Email: [suntao6699@163.com](mailto:suntao6699@163.com)

Feng Wang, M.D., Email: [nxwang@163.com](mailto:nxwang@163.com),

Zhe Jin. Ph.D., Email: [zhe.jin@mcb.uu.se](mailto:zhe.jin@mcb.uu.se).

**Materials and methods**

**Electrode implantation, spontaneous seizures, video/EEG monitoring, recording and analysis**

Using the aseptic technique, a midline incision was made along the anterior-posterior axis, followed by the blunt separation of the subcutaneous fascia to expose the skull. Three holes were drilled, one placed 1.5 mm anterosuperior (left) to the bregma, one placed 3 mm posteroinferior (right) to the bregma, and the other placed 3 mm posteroinferior (left) to the bregma as a fixed electrode, each being 1.5 mm lateral to the central sulcus. Small cranial holes were created with Mini Handheld Skull Drill (#78001; RWD Life Science Co.,Ltd; China), stainless steel microscrews with a diameter of 1.0 mm (#62513; RWD Life Science Co.,Ltd; China) were soldered to 0.5 mm diameter multi-strand copper wires as epidural electrodes, and fixed to the skull with dental acrylic cement. Electrode impedances were typically no more than 10 kΩ. After the placement of electrodes, the skin was closed with suture and the animals was subcutaneously administered with 300 µL of 10% glucose, followed by recovering normal body temperature for 1 hour on a feedback-controlled heat blanket (#TR-200; Safebio; China). The modeling began 7 days after the electrode implantation, EEG patterns were initially evaluated to determine whether anesthesia-related changes were still present. An acquiring and processing system of biomedical signals (#BL-420N; Techman Software; China) was employed for video EEG recording, and the baseline EEG was established. The low-frequency filter was set at 1 Hz, the high-frequency filter was set at 70 Hz and a 50 Hz trap filter was used to reduce line noise if necessary. Seizure-related spike wave discharges (SWDs) were identified by their large amplitude with or without behavioral changes. The peak amplitude of the spike was marked and subsequently visually confirmed. According to our observation, seizure behaviors were graded from stage 1 to 6 based on the modified Racine score^1-3^: (1) normal baseline or freezing; (2): whisker trembling, facial jerking expressed with the nose, sudden behavioral arrest, or motionless staring; (3): neck jerks or oro-facial seizure; (4): rearing and falling, clonic seizures (sitting); (5): continuous rearing or falling seizures & wild jumping; (6): severe generalized tonic clonic seizures (GTCSs) and death.


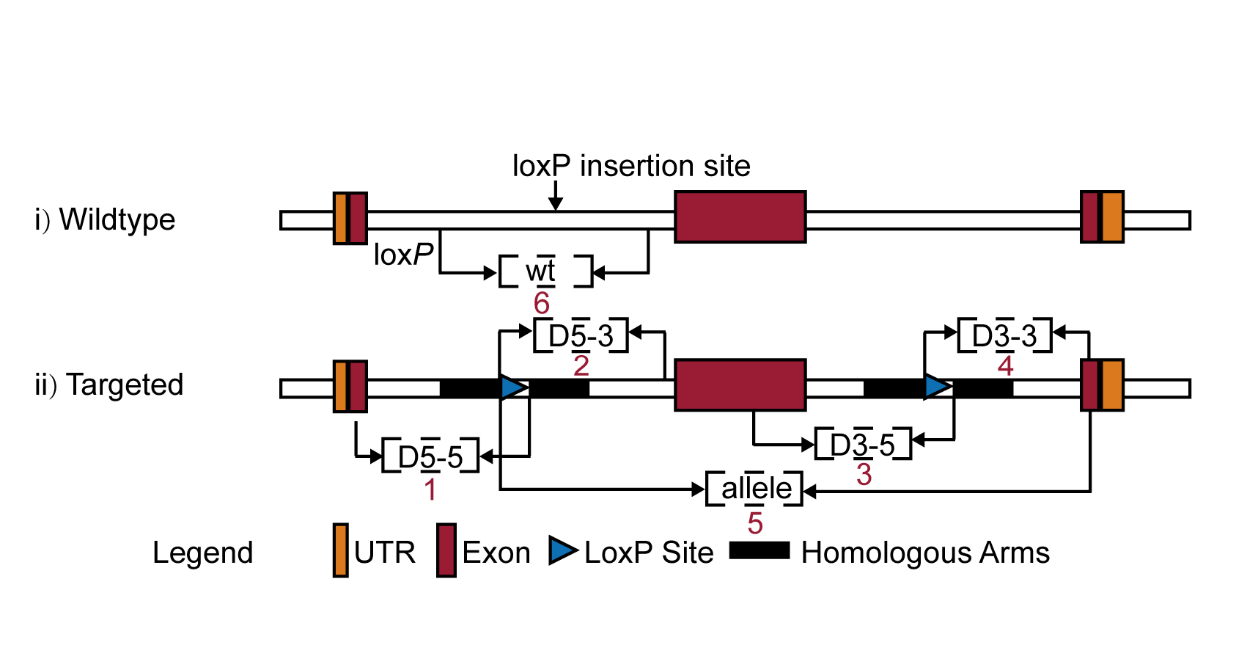


**Fig. S1 Schematic design of primers to identify *Gabrg2* flox mice.** For F_0_ & F_1_ generations, six-pair primers were used for detecting the site of D5-5, D5-3, D3-5, D3-3, as well as D3 allele or D5 allele, and PCR products were then sequenced. For the offspring of F_1_, the primer of 5' preliminary screening probe or 3' preliminary screening probe, D5-5 and D3-3 were used, and no sequencing of PCR product was required.


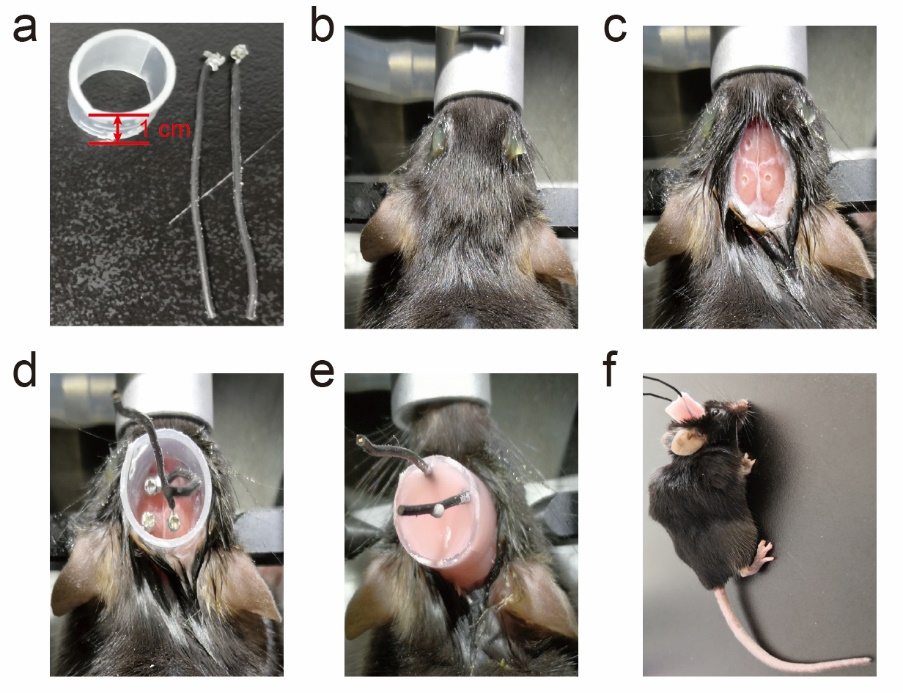


**Fig. S2 The electroencephalography (EEG) electrode implantion surgery. a** A section (1 cm in height) was cut from a 1.5 ml microcentrifugy tube and used as a fixation apparatus to enhance the stability of electrodes and reduce the injury of dental cement to the skin and eyes around the incision. Epidural electrodes with stainless steel screws and multi-strand copper wires were prepared. **b** Maintenance of anesthesia with 2% isoflurane, and prevention of retinitis with erythromycin ointment during the operation. **c** An incision about 1.5 cm was made along the midline after skin disinfection to expose the skull. **d** Three holes were drilled through the skull to dura, one placed 1-2 mm left anterosuperior to the Bregma and the other two placed 2-3 mm posterior to the bregma, each being 2-3 mm lateral to the central sulcus. **e** Two epidural electrodes and one stainless steel screw were carefully implanted, and the prepared sleeve was also fixed to the skull. **f** Electrodes, screw and sleeve were fixed on the skull by dental cement mixed with methyl methacrylate.


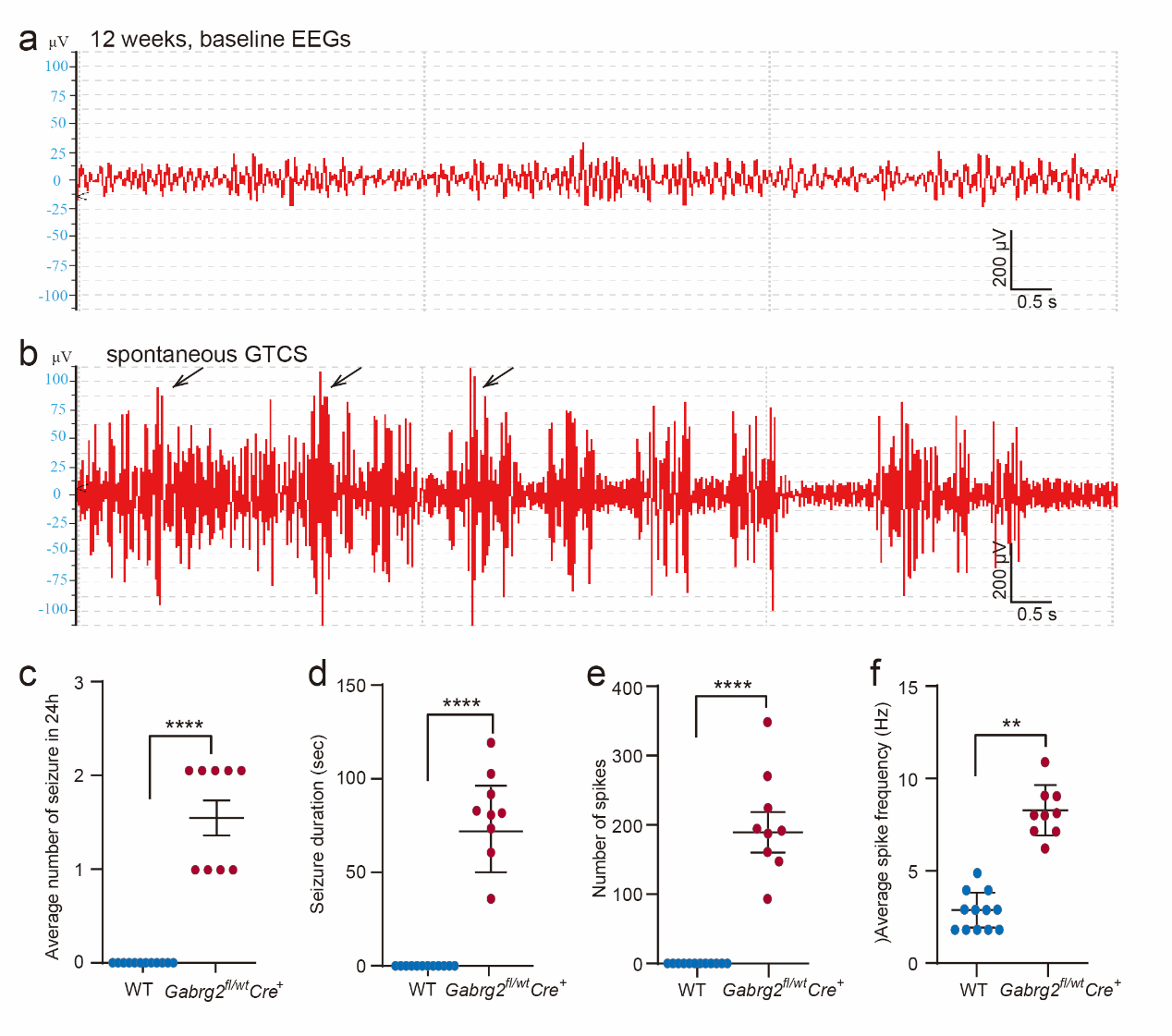


**Fig. S3 Electroencephalography (EEG) in *Gabrg2^fl/wt^Cre^+^* mice at normal body temperature.** **a** A representative 3 s -trace of baseline on intracranial EEG activity from one 8–16 weeks *Gabrg2^fl/wt^Cre^+^* mouse. The amplitude of baseline EEGs in *Gabrg2^fl/wt^Cre^+^* mice was approximately 25–30 μV. **b** EEG recordings showing the generalised tonic-clonic seizures associated with epileptiform activity. A representative intracranial EEG recording of spike-wave discharges during spontaneous myoclonic jerks in an adult *Gabrg2^fl/wt^Cre^+^* mouse (black arrow). **c** Statistical analyses of the average number of seizures (*P* < 0.0001) and (**d**) the seizure duration (*P* < 0.0001) in 24 h for both groups. **e** The total occurrences of spikes were significantly greater in *Gabrg2^fl/wt^Cre^+^* mice than in the WT group (*P* < 0.001). **f** The average spike frequency in 24 h EEG recordings were significantly higher in adult *Gabrg2^fl/wt^Cre^+^* (n = 9) than WT (n = 12) mice (*P* < 0.001). Data are shown as mean ± standard error of mean. ***P* < 0.01, *****P* < 0.0001 vs WT, *t*-test (two-tailed).

**
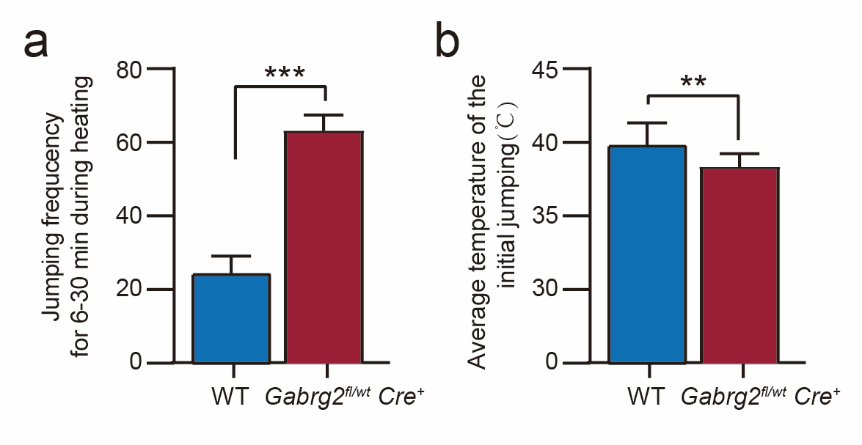
**

**Fig. S4 The number of jumping frequencies and the average temperature of the initial jumping were differentially expressed in *Gabrg2^fl/wt^Cre^+^* mice and WT mice during the heating process. a** The number of jumping frequencies was higher in *Gabrg2^fl/wt^Cre^+^* mice than in WT mice during the temperature elevation seizure iduced (n = 24 for WT and 21 for *Gabrg2^fl/wt^Cre^+^* mice). **b** The average temperature of the initial jumping of *Gabrg2^fl/wt^Cre^+^* mice was lower than that of WT mice (n = 24 for WT and 21 for *Gabrg2^fl/wt^Cre^+^* mice). Data are shown as mean ± standard error of mean. ***P* < 0.01, ****P* < 0.0001 vs WT, *t*-test (two-tailed).

| **Table S1 Primer information for *Gabrg2* flox mouse identification** | | | | |
| --- | --- | --- | --- | --- |
| No. | Primer Name | Sequence | Expected Band Seize | Primer illustration |
| 1 | Gabrg2-ssDNA-5wt-tF1 | GATAAATGGTTGGCTCTAGC | fl=248 bp wt=158 bp | 5' preliminary screening probe Neo-3F |
|  | Gabrg2-ssDNA-5wt-tR1 | ATTAGATTCGCTCCCAACTCC |  |  |
| 2 | Gabrg2-ssDNA-3wt-tF1 | GGGTACATTCACTTATAGAACAACC | fl=294 bp wt=201 bp | 3'preliminary screening probe ZMK2F4 |
|  | Gabrg2-ssDNA-3wt-tR1 | CCAACATTAAGCCTTATGATATTCC |  |  |
| 3 | Gabrg2-ssDNA-5tF1 | CTGGAGTGCTGATAGTAGTGAAAGG | fl=534 bp wt=none | 5'（D5-5） |
|  | Common-En2R | CCAACTGACCTTGGGCAAGAACAT |  |  |
| 4 | Zmk-2F4 | GCATCGCATTGTCTGAGTAGGTG | fl=588 bp wt=none | 3'（D3-3） |
|  | Gabrg2-ssDNA-3tR1 | GACCTACTGTATGCTGCAACTGTG |  |  |
| 5 | Gabrg2-ssDNA-5tF1 | CTGGAGTGCTGATAGTAGTGAAAGG | fl=1962 bp wt=none | 5' allele （D3-5） |
|  | LAR3 | CACAACGGGTTCTTCTGTTAGTCC |  |  |
| 6 | Neo-3F | TCTGAGGCGGAAAGAACCAG | fl=2013 bp wt=none | 3' allele （D5-3） |
|  | Gabrg2-ssDNA-3tR1 | GACCTACTGTATGCTGCAACTGTG |  |  |
| Fl = flox; wt = wild type | | | | |

| **Table S2 Primer information for *Gabrg2^fl/wt^Cre+* mouse identification** | | | | |
| --- | --- | --- | --- | --- |
| No. | Primer Name | Sequence | Expected Band Seize | Primer illustration |
| 1 | Gabrg2-ssDNA-5wt-tF1 | GATAAATGGTTGGCTCTAGC | fl=248 bp wt=158 bp  Null=none | Heterozygous or homozygous identification |
|  | Gabrg2-ssDNA-5wt-tR1 | ATTAGATTCGCTCCCAACTCC |  |  |
| 2 | Zmk-2F4 | GCATCGCATTGTCTGAGTAGGTG | fl=588 bp wt=none  Null=none | 3' loxp identification |
|  | Gabrg2-ssDNA-3tR1 | GACCTACTGTATGCTGCAACTGTG |  |  |
| 3 | Cre-up | GCCTGCATTACCGGTCGATGC | T：481 bp | Detect Emxl-Cre |
|  | Cre-low | CAGGGTGTTATAAGCAATCCC |  |  |
| 4 | Gabrg2-null-tF1 | ATAGCTGTGACGACGACGGGTG | fl=1852 bp wt=1669 bp  Null=424 bp | Null identification |
|  | Gabrg2-null-tR1 | CCCTCCTGTGAGTGAGGTTACTTC |  |  |
| Null = flox region has been deleted; T = transgenic | | | | |

| **Table S3 GABRG2 mutations associated or likely associated with various epilepsy phenotypes** | | | | | | | | | | | |
| --- | --- | --- | --- | --- | --- | --- | --- | --- | --- | --- | --- |
| Patient or mouse model | Phenotype(s) | | | | | | | | | | Reference(s) |
|  | FS | FS+ | CAE | CS | TS | GCTS | GEFS+ | DS | EE | other |  |
| Human | √ | √ | √ | √ | √ | √ | √ | √ | √ | √ | 4,5,6,7,8,9,10 |
| *Gabrg2^fl/wt^Cre^+^* (KO) | √ | √ | ND | √ | √ | √ | √ | ND | ND | √ | the present work |
| *Gabrg2^+/-^* (KO) |  |  | √ |  |  |  |  |  |  |  | 11,12 |
| *Gabrg2^+/R43Q^* (KI) | √ |  | √ |  |  |  | √ |  |  |  | 13,14 |
| *Gabrg2 ^IVS6+2T→G^* (KI) |  |  | √ |  |  |  |  |  |  |  | 15 |
| *Gabrg2^+/Q390X^* (KI) | √ | √ | ND | √ | √ | √ | √ | √ | √ | √ | 1,12,16 |
| FS: febrile seizures; FS+: febrile seizure plus; CAE: childhood absence epilepsy; CS: clonic seizures; TS: tonic seizures; GCTS: generalised tonic-clonic seizures; GEFS+: genetic epilepsy with febrile seizure plus; DS: Dravet syndrome; EE: epileptic encephalopathy; ND: not determined; KO; knock out; KI: knock in. | | | | | | | | | | | |

**References**

1. Warner TA, Liu Z, Macdonald RL & Kang JQ. Heat induced temperature dysregulation and seizures in Dravet Syndrome/GEFS+ Gabrg2(+/Q390X) mice. *Epilepsy Res* **134,** 1-8 (2017)
2. Van Erum J, Van Dam D & De Deyn PP. PTZ-induced seizures in mice require a revised Racine scale. *Epilepsy Behav* **95,** 51-55 (2019)
3. Lüttjohann A, Fabene PF & van Luijtelaar G. A revised Racine's scale for PTZ-induced seizures in rats. *Physiol Behav* **98,** 579-586 (2009)
4. Scheffer IE & Berkovic SF. Generalized epilepsy with febrile seizures plus. A genetic disorder with heterogeneous clinical phenotypes. *Brain* **120**, 479-490 (1997)
5. Wallace RH, Marini C, Petrou S, Harkin LA, Bowser DN, Panchal RG, et al. Mutant GABA(A) receptor gamma2-subunit in childhood absence epilepsy and febrile seizures. *Nat Genet* **28,** 49-52 (2001)
6. Shen D, Hernandez CC, Shen W, Hu N, Poduri A, Shiedley B, et al. De novo GABRG2 mutations associated with epileptic encephalopathies. *Brain* **140,** 49-67 (2017)
7. Boillot M, Morin-Brureau M, Picard F, Weckhuysen S, Lambrecq V, Minetti C, et al. Novel GABRG2 mutations cause familial febrile seizures. *Neurol Genet* **1,** e35 (2015)
8. Harkin LA, Bowser DN, Dibbens LM, Singh R, Phillips F, Wallace RH, et al. Truncation of the GABA(A)-receptor gamma2 subunit in a family with generalized epilepsy with febrile seizures plus. *Am J Hum Genet* **70,** 530-536 (2002)
9. Kananura C, Haug K, Sander T, Runge U, Gu W, Hallmann K, et al. A splice-site mutation in GABRG2 associated with childhood absence epilepsy and febrile convulsions. *Arch Neurol* **59,** 1137-1141 (2002)
10. Butilă AT, Zazgyva A, Sin AI, Szabo ER & Tilinca MC. GABRG2 C588T gene polymorphisms might be a predictive genetic marker of febrile seizures and generalized recurrent seizures: a case-control study in a Romanian pediatric population. *Arch Med Sci* **14,** 157-166 (2018)
11. Reid CA, Kim T, Phillips AM, Low J, Berkovic SF, Luscher B, et al. Multiple molecular mechanisms for a single GABAA mutation in epilepsy. *Neurology* **80,** 1003-1008 (2013)
12. Warner TA, Shen W, Huang X, Liu Z, Macdonald RL & Kang JQ. Differential molecular and behavioural alterations in mouse models of GABRG2 haploinsufficiency versus dominant negative mutations associated with human epilepsy. *Hum Mol Genet* **25,** 3192-3207 (2016)
13. Tan HO, Reid CA, Single FN, Davies PJ, Chiu C, Murphy S, et al. Reduced cortical inhibition in a mouse model of familial childhood absence epilepsy. *Proc Natl Acad Sci U S A* **104,** 17536-17541 (2007)
14. Hill EL, Hosie S, Mulligan RS, Richards KL, Davies PJ, Dube CM, et al. Temperature elevation increases GABA(A) -mediated cortical inhibition in a mouse model of genetic epilepsy. *Epilepsia* **52,** 179-184 (2011)
15. Tian M & Macdonald RL. The intronic GABRG2 mutation, IVS6+2T->G, associated with childhood absence epilepsy altered subunit mRNA intron splicing, activated nonsense-mediated decay, and produced a stable truncated gamma2 subunit. *J Neurosci* **32,** 5937-5952 (2012)
16. Kang JQ, Shen W, Zhou C, Xu D & Macdonald RL. The human epilepsy mutation GABRG2(Q390X) causes chronic subunit accumulation and neurodegeneration. *Nat Neurosci* **18,** 988-996 (2015)
