# Supplementary figures and images for: Neocortex- and hippocampus-specific deletion of Gabrg2 causes temperature-dependent seizures in mice
Source: Cell Death Dis. 2021 May 28;12(6):553. doi: 10.1038/s41419-021-03846-x (PMC8163876; doi:10.1038/s41419-021-03846-x)

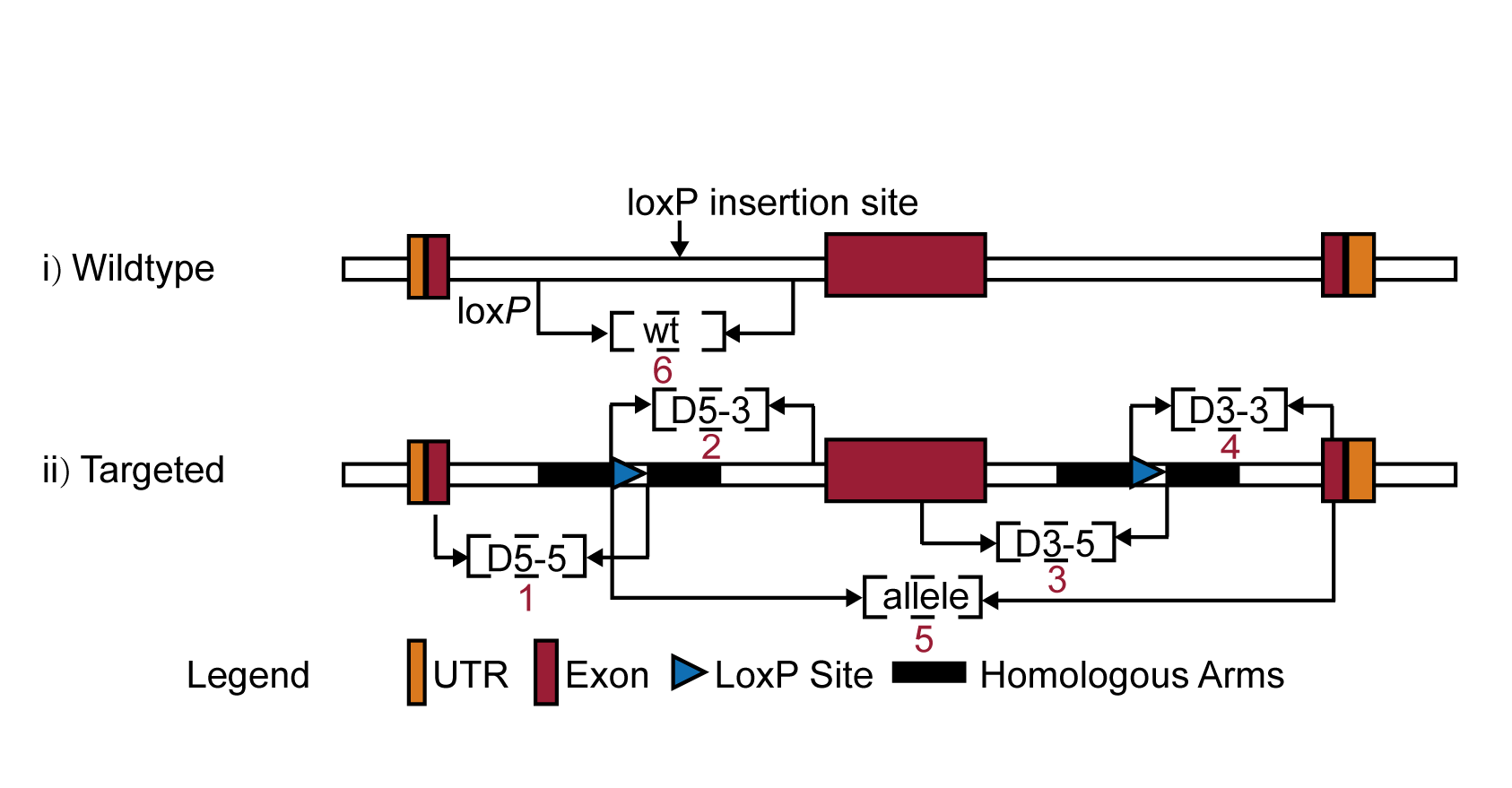

Supplement: Supplementary file 2 — SI Fig.1 [file 41419_2021_3846_MOESM2_ESM.tif]

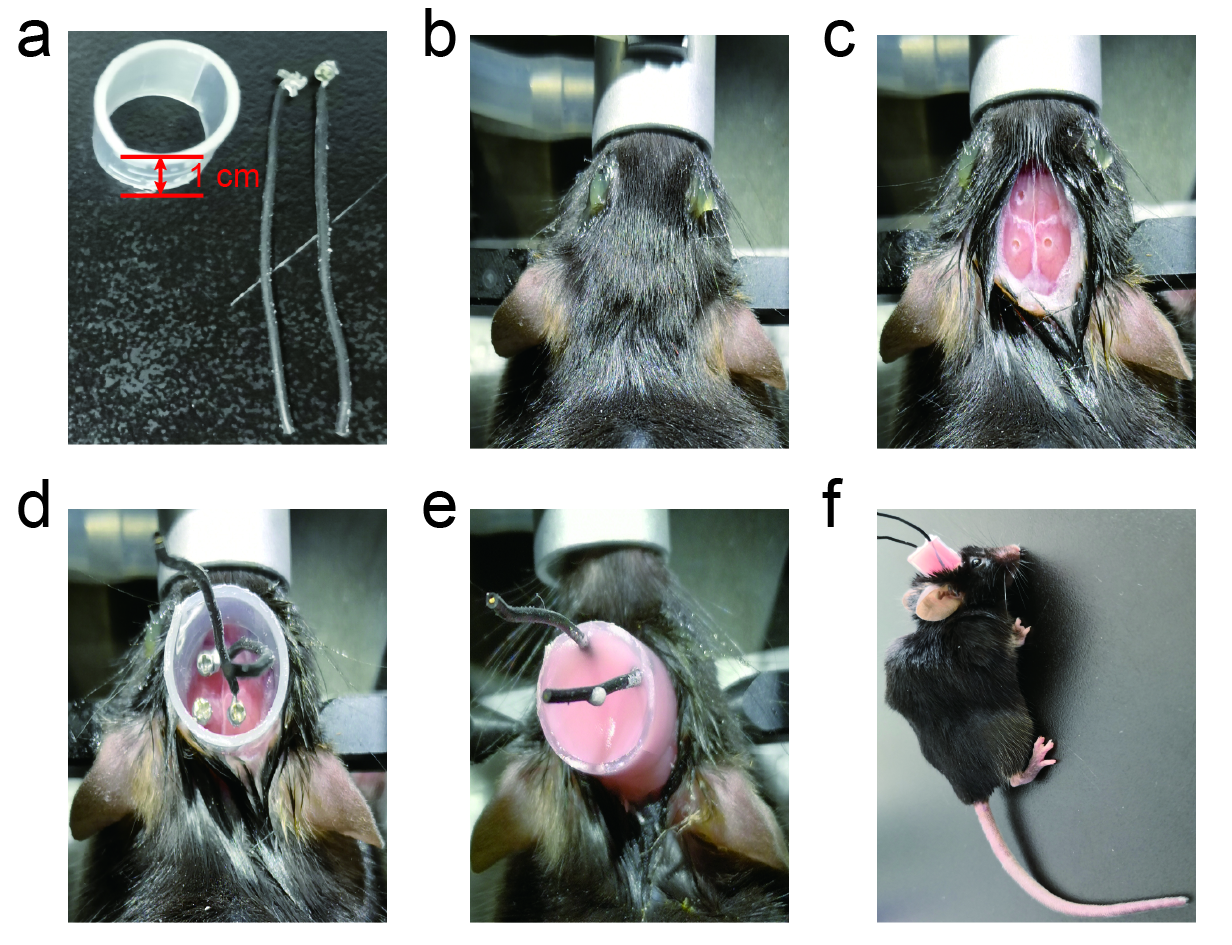

Supplement: Supplementary file 3 — SI Fig.2 [file 41419_2021_3846_MOESM3_ESM.tif]

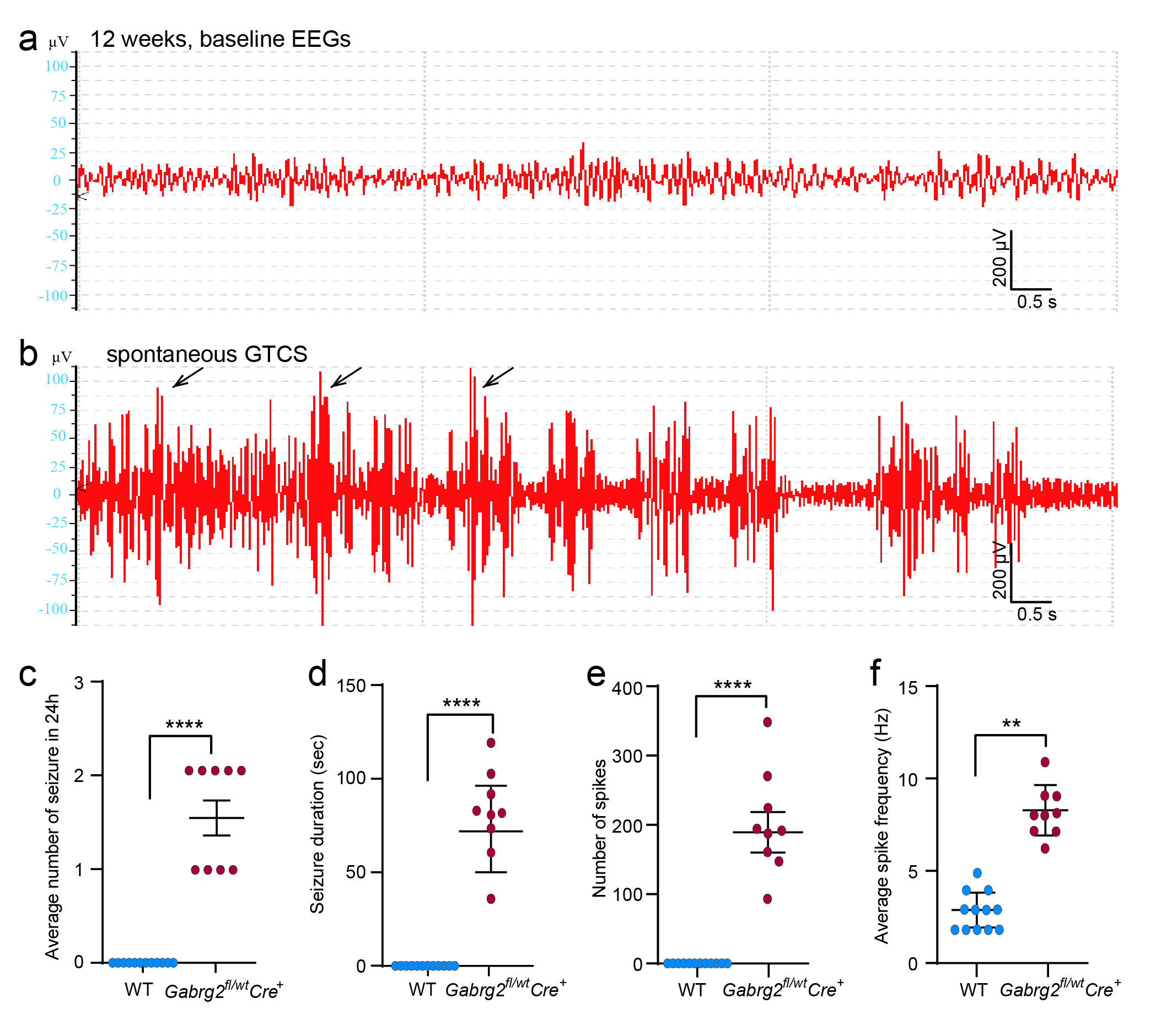

Supplement: Supplementary file 4 — SI Fig.3 [file 41419_2021_3846_MOESM4_ESM.tif]

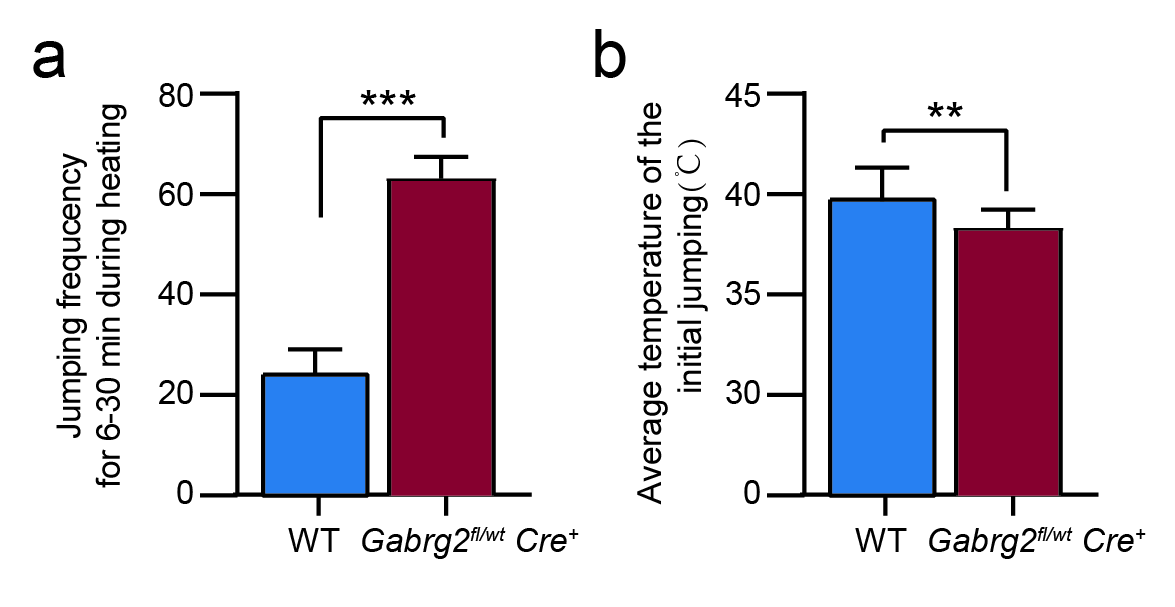

Supplement: Supplementary file 5 — SI Fig.4 [file 41419_2021_3846_MOESM5_ESM.tif]
